# Single-cell compendium of muscle microenvironment in peripheral artery disease reveals altered endothelial diversity and LYVE1^+^ macrophage activation

**DOI:** 10.1038/s44161-025-00709-y

**Published:** 2025-09-15

**Authors:** Guillermo Turiel, Thibaut Desgeorges, Evi Masschelein, Zheng Fan, David Lussi, Christophe M. Capelle, Giulia Bernardini, Raphaela Ardicoglu, Katharina Schönberger, Manuela Birrer, Sandro F. Fucentese, Jing Zhang, Daniela Latorre, Stephan Engelberger, Katrien De Bock

**Affiliations:** 1 Laboratory of Exercise and Health, Department of Health Sciences and Technology, Swiss Federal Institute of Technology (ETH), Zurich, Switzerland; 2 Institute of Anatomy, University of Zurich, Zurich, Switzerland; 3 Institute of Microbiology, Department of Biology, Swiss Federal Institute of Technology (ETH), Zurich, Switzerland; 4 Zentrum für Gefässchirurgie, Kantonsspital Aarau, Aarau, Switzerland; 5 Vascular Center, Department of General, Visceral and Vascular Surgery, Kantonsspital Baden, Baden, Switzerland; 6 Department of Orthopedics, University Hospital Balgrist, University of Zurich, Zurich, Switzerland; 7 Institute of Experimental Neurology, Division of Neuroscience, IRCCS Ospedale San Raffaele (OSR), Milan, Italy

**Keywords:** Peripheral vascular disease, Preclinical research, RNA sequencing

## Abstract

Peripheral artery disease (PAD) results from atherosclerosis and chronic narrowing of lower limb arteries, leading to decreased muscle perfusion. Current treatments are suboptimal, partly due to limited understanding of PAD muscle pathology. Here we used single-cell RNA sequencing and spatial transcriptomics to analyze the composition of the muscle microenvironment in non-ischemic patients and patients with PAD. We identified *ATF3/ATF4*^+^ endothelial cells (ECs) that exhibit altered angiogenic and immune regulatory profiles during PAD and confirmed that ATF4 signaling in ECs is required for effective ischemia recovery. In addition, capillary ECs display features of endothelial-to-mesenchymal transition. Furthermore, *LYVE1*^hi^MHCII^low^ macrophages are the dominant macrophage population in human muscle, adopting a more pro-inflammatory profile during PAD. Finally, we analyzed alterations in intercellular communication within the muscle microenvironment during PAD and confirmed that EC-derived factors can influence macrophage polarization. This dataset deeply characterizes the PAD muscle microenvironment and provides a resource for exploration of targeted therapies.

## Abstract (web summary)

Using single-cell and spatial transcriptomics on muscle samples from non-ischemic patients and patients with peripheral artery disease (PAD), Turiel et al. identify cellular and molecular changes in the muscle microenvironment during PAD, focusing on endothelial cell–macrophage interactions.

## Main

Peripheral artery disease (PAD) is caused by atherosclerosis and chronic narrowing of lower limb arteries, leading to decreased muscle perfusion and oxygenation. Cardiovascular risk factors, such as hypertension, hyperlipidemia and diabetes mellitus, are important driving risk factors of PAD, and patients often present other cardiovascular diseases^1^. Patients with PAD suffer from pain and fatigue in the limb muscles due to exercise-induced ischemia, which resolves after a short period of rest—a syndrome termed intermittent claudication^2,3^. If left untreated or in more severe stages, persistent lack of limb perfusion can lead to critical limb ischemia (CLI), which comprises rest pain, tissue loss and necrosis and ulcerations, which often progresses to amputation^1^. Current guidelines for treating PAD target large atherosclerotic lesions in the feeding arteries, but long-term clinical outcomes have been suboptimal, and many patients suffer from adverse events^4^.

One potential reason for this might be that there is underlying skeletal muscle and vascular pathology in PAD^3^. Indeed, microvascular dysfunction has been reported, with abnormal microvascular architecture and fibrosis, and a few studies recently reported partial endothelial-to-mesenchymal transition (EndoMT) in patients with PAD^5,6^ and capillaries of end-stage patients^6^. In addition, impaired vasoreactive responses might impair oxygen and nutrient delivery into the ischemic muscle^7^. Interestingly, some^8–11^ but not all^12,13^ studies observed increased vascular density. Although the discrepancy underlying these conflicting observations is unclear, persistent ischemia in patients with PAD despite increased vascularization nonetheless suggests that an active angiogenic response does not resolve tissue ischemia.

Studies using preclinical muscle injury models showed that microvascular endothelial cells (ECs) are not only essential for inducing muscle revascularization; ECs also steer muscle regeneration by interacting with other cells in their microenvironment. ECs activate muscle stem cells^14^, control vascular tone through interaction with smooth muscle cells (SMCs)^15^ and control the entry of immune cells into the tissue, control their differentiation into specialized immune effectors as well as (co-)define their functional properties^16,17^. However, how ECs interact with other cell types and how they might contribute to skeletal muscle pathology in PAD remain largely unknown.

Macrophages are key regulators of muscle regeneration^18^ but also stimulate muscle angiogenesis as well as arteriogenesis in preclinical PAD models^16,19^. The genetic and functional contribution of macrophages to skeletal muscle pathology in PAD is, however, unclear. Higher levels of inflammatory cytokines correlated with shorter-onset claudication pain^20^, but assessments of macrophage numbers and properties in skeletal muscle showed conflicting results, with one study showing a majority of pro-inflammatory CD80^+^ macrophages^21^, whereas others reported higher CD11b^+^CD206^+^ regenerative macrophages^22^.

In the present study, we performed a thorough transcriptomic and functional characterization of the mononuclear cell composition in skeletal muscle during PAD. We aimed to better understand the contribution of the muscle microenvironment to PAD.

## Results

### Single-cell RNA sequencing reveals a high degree of cellular heterogeneity in human skeletal muscle

**Fig. 1:**
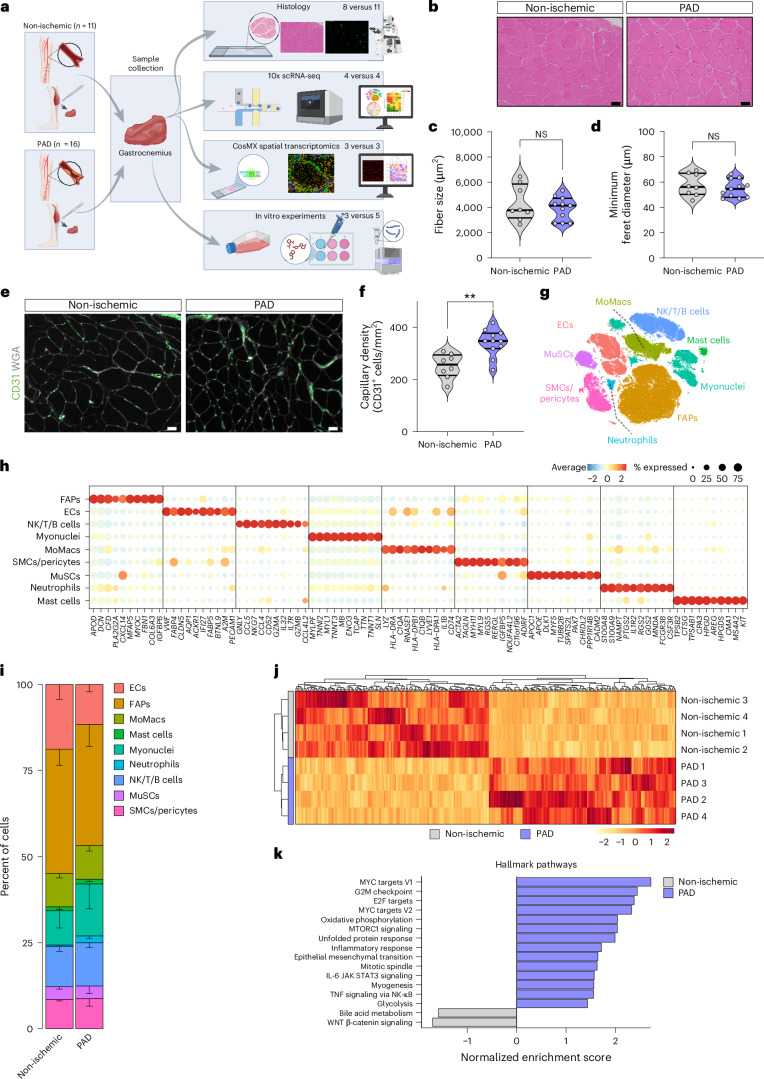
scRNA-seq reveals cell heterogeneity in PAD. **a**, Graphical abstract of the experimental approach used in this study. **b**, Representative images of H&E-stained sections of gastrocnemius muscle from non-ischemic patients (*n* = 8) and patients with PAD (*n* = 11). Scale bars, 50 µm. **c**,**d**, Quantification of fiber size in µm^2^ (**c**) and minimum feret diameter in µm (**d**) from **b**. **e**, Representative images of gastrocnemius muscle from non-ischemic (*n* = 8) and PAD (*n* = 11) stained for the EC marker (CD31, green) and WGA (white). Scale bars, 50 µm. **f**, Quantification of capillary density from **e** measured as the number of CD31^+^ cells per mm^2^. **g**, t-SNE plot of cell populations identified from human gastrocnemius muscle, color coded by the identified populations. **h**, Dot plot of centered log count values from cell-type-specific marker genes. Color and size of the dots indicate the centered log count value and the proportion of cells that express the gene, respectively. **i**, Stacked bar plots showing cluster percentage from non-ischemic patients (*n* = 4) and patients with PAD (*n* = 4), color coded by cluster. Each stack represents mean − s.e.m. **j**, Heatmap of centered normalized values from top high variable genes (adjusted *P* < 0.05) in each patient based on pseudobulk analysis between non-ischemic and PAD conditions; color indicates the centered normalized values. **k**, Bar plots showing normalized enrichment score of significant (adjusted *P* < 0.05) hallmark pathways (MSigDB) from GSEA analysis on the pseudobulk dataset; color indicates the condition. Each dot represents a single patient in **c**, **d** and **f**. Student’s *t*-test (two-tailed, unpaired, parametric, NS > 0.05, **P < 0.01) was used in **c**, **d** and **f**. Wald test (as implemented in the DESeq2 package) was used in **j**. Adaptive multilevel Monte Carlo scheme (as implemented in the fgsea package) was used in **k**. *P* = 0.4535 (**c**), *P* = 0.4585 (**d**), *P* = 0.0012 (**f**). NK, natural killer; NS, non-significant. Panel **a** created with BioRender.com. Source data

To investigate the cellular landscape of patients with PAD, we collected gastrocnemius muscle samples from either individuals undergoing PAD bypass surgery (PAD) or comorbidity-matched non-ischemic individuals undergoing lower limb aneurysm surgery (non-ischemic) (Methods, Fig. 1a and Table 1). Non-ischemic patients had a normal ankle-brachial index (ABI) and no clinical symptoms of PAD (no claudication symptoms, Fontaine classification = I) (Table 1). Preoperative angiographic imaging revealed that only patients with PAD presented severe popliteal occlusions (Global Limb Anatomic Staging System (GLASS) grade III).

**Table 1:** Patient demographics

	Non-ischemic	PAD
Number of patients	11	16
Average age (years)	73.36 ± 2.16	73.88 ± 2.12
Average height (cm)	175 ± 2.04	174.69 ± 1.78
Average weight (kg)	78.96 ± 5.52	79.75 ± 3.27
Average BMI (kg m^−2^)	25.65 ± 1.55	26.06 ± 0.85
ABI	1.12 ± 0.06	0.75 ± 0.08**
Fontaine classification I/IIa/IIb/III/IV	11/0/0/0/0	0/0/9/3/4
GLASS stage NA/I/II/III	10/1/0/0	0/0/0/16
scRNA-seq/histology/spatial/in vitro	4/8/3/3^†^	4/11/3/5^†^
Gender (male/female)	11/0	16/0
Smoking (never/current/former)	3/4/4	5/8/3
Obesity (BMI > 30 kg m^−2^)	1 (9.1%)	2 (12.5%)
Diabetes mellitus	3 (27.3%)	7 (43.8%)
Hypertension	9 (81.8%)	13 (81.3%)
Hyperlipidemia	8 (72.7%)	14 (87.5%)
Statins	7 (63.6%)	13 (81.3%)
Antiplatelet agents	10 (90.9%)	11 (68.8%)
Antihypertensive drugs	10 (90.9%)	12 (75.0%)
Antidiabetic agents	3 (27.3%)	7 (43.8%)
Involved side (left/right)	5/6	8/8

Continuous values are presented as mean ± s.e.m. BMI, body mass index.

**P < 0.01, Student’s *t*-test (unpaired, two-tailed).

^†^A subset of histology samples was used for scRNA-seq and/or spatial experiments. In vitro experiments were performed with three PAD samples for bulk RNA-seq and three versus five for conditioned media experiments.

**Extended Data Fig. 1:**
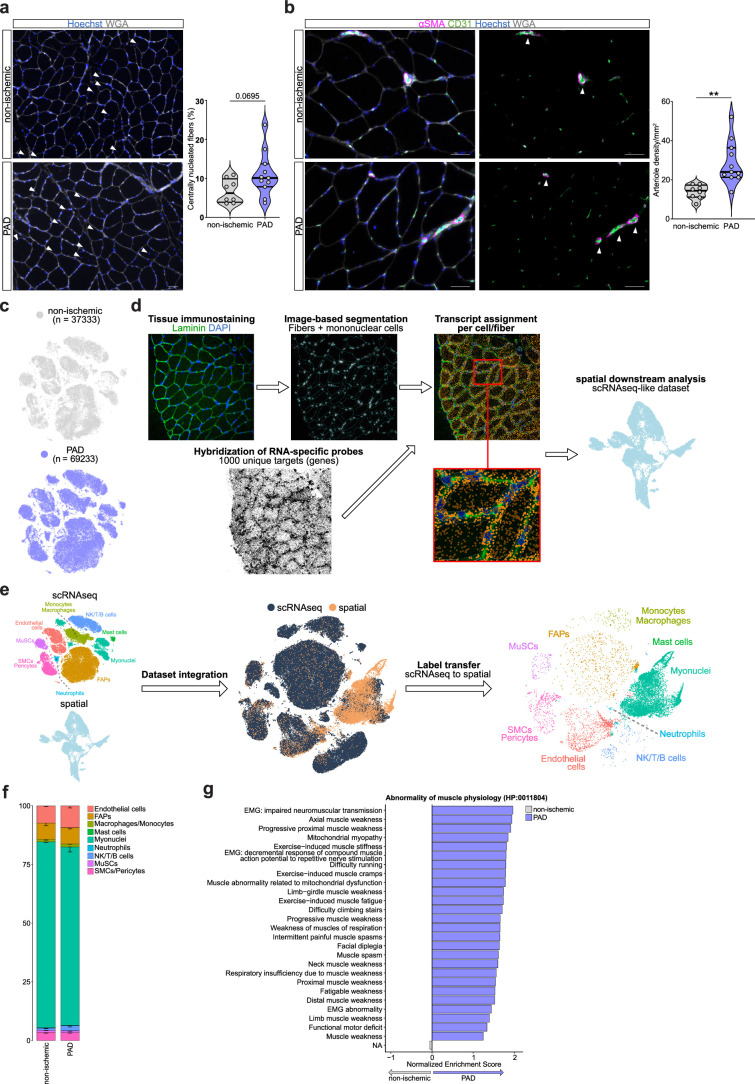
scRNAseq reveals cell heterogeneity in PAD. **a**, Representative images of gastrocnemius muscle from non-ischemic (n = 8) and PAD (n = 11) stained for nuclei (Hoechst, blue) and WGA (white) (Scale bar: 50 µm) and quantification of percentage of centrally nucleated fibers over total fibers. **b**, Representative images of gastrocnemius muscle from non-ischemic (n = 8) and PAD (n = 11) stained for αSMA (magenta), CD31 (green), nuclei (Hoechst, blue) and WGA (white) (Scale bar: 50 µm) and quantification of number of arterioles per mm^2^. **c**, TSNE plot (Fig. 1g) separated by condition, color indicates the condition. **d**, Schematic representation of the pipeline used for generating the spatial transcriptomics dataset (see Methods). **e**, Schematic representation of the pipeline used for transferring cluster labels from scRNAseq to spatial data (see Methods). **f**, Stacked bar plots showing cluster percentage in each condition in the spatial dataset from non-ischemic (n = 3) and PAD (n = 3) patients, color-coded by cluster. Each stack represents mean - SEM. **g**, Bar plots showing Normalized Enrichment Score of significant (adjusted p-value < 0.05) “Abnormality of muscle physiology” pathways (Human Phenotype Ontology) from GSEA analysis on the pseudobulk dataset, color indicates the condition. Each dot represents a single patient in panels **a** and **b**. Student’s t test (two-tailed, unpaired, parametric, ns > 0.05) was used in **a** and **b**. Adaptive multi-level Monte-Carlo scheme (as implemented in fgsea package) was used in g. P = 0.0695 (**a**), P = 0.0019 (**b**). Source data

Neither myofiber size nor diameter was affected in patients with PAD (Fig. 1b–d). In addition, we did not observe muscle necrosis but noticed a small non-significant increase in the percentage of regenerating fibers (Extended Data Fig. 1a) and increased CD31^+^ capillary (Fig. 1e,f) and arteriole (Extended Data Fig. 1b) densities. We next performed single-cell RNA sequencing (scRNA-seq) in a subset of patients (Table 1) and generated a dataset comprising 106,566 cells (2,359 genes per cell). Clustering revealed nine major cell populations (Fig. 1g) that we manually annotated based on the expression of well-known marker genes^23^ (Fig. 1h; fibro-adipogenic progenitors (FAPs), natural killer (NK) cells, monocytes/macrophages (MoMacs), SMCs and muscle stem cells (MuSCs)). We identified the main cell types in skeletal muscle (Fig. 1g,h) and did not detect obvious differences in cell proportions between groups (Fig. 1i and Extended Data Fig. 1c).

We additionally performed single-cell spatial transcriptomics to directly localize scRNA-seq populations (Table 1). We identified muscle fibers and mononuclear cells by combining transcript detection with cell segmentation (Extended Data Fig. 1d) and annotated each cell through integration with the scRNA-seq dataset (Extended Data Fig. 1e). Approximately 75% of the detected cells were fiber derived (Extended Data Fig. 1f), reflecting the predominance of myonuclei in skeletal muscle. Despite these expected differences with the scRNA-seq data, ECs, FAPs and SMCs/pericytes still constituted most of the mononuclear fraction.

We first compared the scRNA-seq patients by pseudobulk analysis, which showed different transcriptomic signatures between groups (Fig. 1j and Supplementary Data 1). Gene set enrichment analysis (GSEA) revealed increased TNF and interleukin 6 (IL-6) signaling as well as inflammatory activation, unfolded protein response and metabolic pathways, whereas WNT signaling and bile acid metabolism were repressed (Fig. 1k). Additionally, GSEA using a gene set collection from Human Phenotype Ontology (‘Abnormality of muscle physiology’, HP:0011804) revealed increased PAD-like clinical symptoms, whereas no term was enriched in non-ischemic patients (Extended Data Fig. 1g).

Next, we performed a comprehensive analysis on ECs, macrophages and SMCs/pericytes because of their crucial role in angiogenesis, inflammation and tissue repair^21^. The data, including all identified cell populations, can be interactively explored at https://shiny.debocklab.hest.ethz.ch/Turiel-et-al/.

### Human skeletal muscle contains *ATF3/ATF4*^+^ endothelial subpopulations with immunoregulatory signatures

**Fig. 2:**
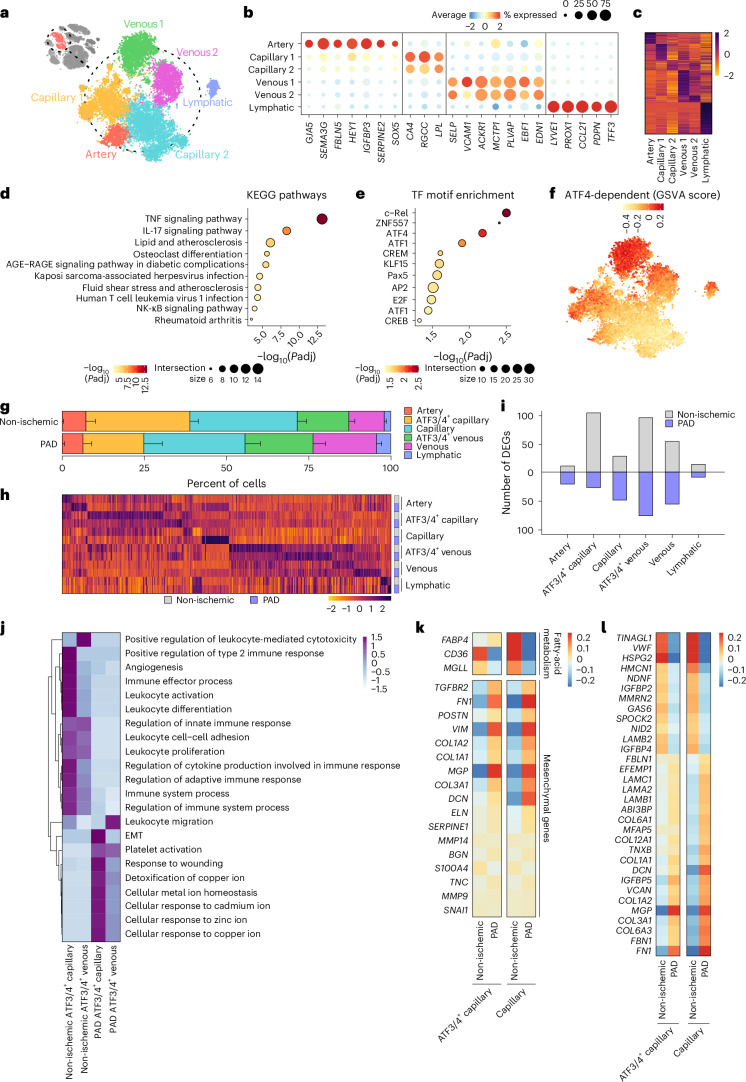
Human skeletal muscle contains several EC subtypes with different functional properties. **a**, t-SNE plot of EC subtypes identified from human gastrocnemius muscle, color coded by the identified subtypes. **b**, Dot plot of centered log count values from EC subtype-specific marker genes. Color and size of the dots indicate the centered log count value and the proportion of cells that express the gene, respectively. **c**, Heatmap of centered log counts of DEGs (adjusted *P* < 0.05) between EC subtypes; color indicates the centered log count value. **d**,**e**, ORA from genes upregulated in venous 1 compared to venous 2 (Extended Data Fig. 2a) using KEGG pathways (**d**) or transcription factor motif enrichment (**e**); color and size of the dots indicate the −log_10_ adjusted *P* value and the number of genes in each category (intersection size), respectively. **f**, t-SNE plot of GSVA scores using the ATF4-dependent gene set from Fan et al.^24^ (Methods), color coded by the GSVA score. **g**, Stacked bar plots showing cluster percentage from non-ischemic patients (*n* = 4) and patients with PAD (*n* = 4), color coded by cluster. Each stack represents mean − s.e.m. **h**, Heatmap of centered log counts of DEGs (adjusted *P* < 0.05) specific for each EC subtype in each condition; color indicates the centered log count value. **i**, Bar plots showing the number of DEGs for each EC subtype in each condition; color indicates the condition. **j**, Heatmap of centered values from ORA over the DEGs in *ATF3/ATF4*^+^ venous and capillary ECs between conditions; color indicates the centered values. **k**,**l**, Heatmap of centered log count values of fatty acid metabolism and mesenchymal markers as defined in Tombor et al.^32^ (**k**) and core matrisome genes as defined in Hynes et al.^33^ (**l**); color indicates the centered values. Wilcoxon rank-sum test (as implemented in the Seurat package) was used in **c** and **h**. Fisher’s one-tailed test (as implemented in g:Profiler) was used in **d** and **e**. *P*adj, adjusted *P* value; TF, transcription factor. Source data

**Extended Data Fig. 2:**
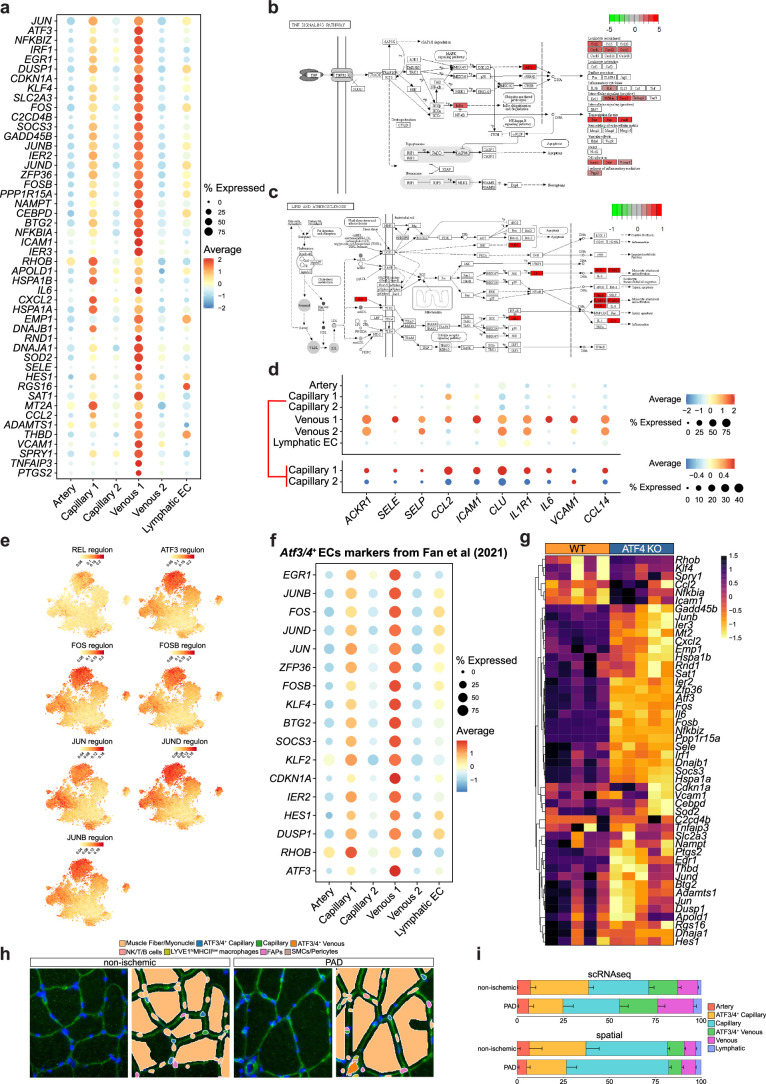
A subpopulation of ECs shows an *ATF3/ATF4* transcriptomic signature. **a**, Dot plot of centered logcounts values from DEGs (adjusted p-value < 0.05 and log2FC > 1) upregulated in Venous 1 compared to Venous 2. **b-c**, Schematic view of TNF signaling (**b**) and lipid and atherosclerosis (**c**) pathways from KEGG (see Fig. 2d). Color indicates the log2FC of genes upregulated in Venous 1 compared to Venous 2. **d**, Dot plot of centered logcounts values from immunomodulatory ECs (IMECs) markers obtained from Alnaqbi et al. **e**, TSNE plots showing the SCENIC regulon activity score for REL and ATF4-dependent transcription factors. Color indicates the regulon activity score. **f**, Dot plot of centered logcounts values from mouse *ATF3/ATF4*^+^ Capillary marker genes obtained from Fan et al. **g**, Heatmap of centered normalized values of genes from panel **a** in the bulk RNAseq experiment of muscle ECs between WT and EC-specific ATF4 KO mice from Fan et al. Color indicates the centered normalized value. **h**, Images of non-ischemic and PAD muscle samples annotated in the spatial dataset after transferring cell labels from the scRNAseq dataset (see Extended Data Fig. 1e). Stained for laminin (green) and cell nuclei (blue, DAPI). Legend only shows visible cell types in the images. **i**, Stacked bar plots showing cluster percentage in each condition in the scRNAseq (top, non-ischemic n = 4, PAD n = 4) and spatial (bottom, non-ischemic n = 3, PAD n = 3) datasets, color-coded by cluster. Each stack represents mean - SEM. Wilcoxon Rank Sum test (as implemented in Seurat package) was used in **a**. Source data

We selected ECs from the complete dataset and detected six different populations, which we annotated using described marker genes^24,25^ (Fig. 2a,b). Each population presented specific signatures, demonstrating clustering robustness (Fig. 2c and Supplementary Data 1). We identified two distinct populations of venous and capillary ECs that expressed similar markers (Fig. 2b) but showed different transcriptomic signatures (Fig. 2a,c). Differential expression analysis between both venous clusters detected a set of genes specifically enriched in venous 1 and capillary 1 populations (Extended Data Fig. 2a). Overrepresentation analysis (ORA)^26^ revealed that these genes are associated with recruitment and functional modulation of immune cells by ECs, including TNF, IL-17 and NF-κB pathways^17,27,28^, as well as a lipid and atherosclerosis signaling (Fig. 2d). A deeper analysis of these pathways revealed that they are involved in leukocyte recruitment and activation, cell adhesion and cytokine signaling (Extended Data Fig. 2b,c). Recent studies^29^ have identified immunomodulatory endothelial cells (IMECs), and the genes that define IMECs^29^ were enriched in venous 1 ECs (Extended Data Fig. 2d). We also observed higher expression in capillary 1 than in capillary 2 (Extended Data Fig. 2d, bottom panel), suggesting that both populations are ECs with immunoregulatory features.

ORA for transcription factor motif enrichment identified c-Rel, ZNF557 and ATF4 as top transcription factors regulating the genes enriched in both venous 1 and capillary 1 populations (Fig. 2e and Extended Data Fig. 2a). We also applied SCENIC^30^, which generates a ‘TF regulon activity score’ based on the co-expression of transcription factors and their target genes. SCENIC did not detect ZNF557 as an active transcription factor, due to its few detected target genes (intersection size in Fig. 2e), and c-Rel activity was relatively weak (Extended Data Fig. 2e). By contrast, ATF4-dependent transcription factors showed high and specific activity in venous 1 and capillary 1 clusters (Extended Data Fig. 2e), so we focused on *ATF3/ATF4*. We previously identified *Atf3/Atf4*^+^ muscle ECs in mouse that control exercise-induced angiogenesis^24^. The marker genes defining *Atf3/Atf4*^+^ ECs were highly enriched in both venous 1 and capillary 1 human clusters (Extended Data Fig. 2f). Conversely, the marker genes from the human populations (Extended Data Fig. 2a) were also controlled by ATF4 in ECs (Extended Data Fig. 2g). We also used an ATF4-dependent gene signature to perform gene set variation analysis (GSVA)^31^, which calculates a score for each cell based on the expression levels of an input gene signature. The ATF4-dependent GSVA score was enriched in venous 1 and capillary 1 clusters (Fig. 2f), and we, therefore, annotated these populations as *ATF3/ATF4*^+^ venous ECs and *ATF3/ATF4*^+^ capillary ECs. The fraction of *ATF3/ATF4*^+^ capillaries was reduced in PAD when compared to non-ischemic samples (Fig. 2g). We also identified all EC subtypes in the spatial dataset and found very consistent cluster proportions between both technologies (Extended Data Fig. 2h,i).

**Extended Data Fig. 3:**
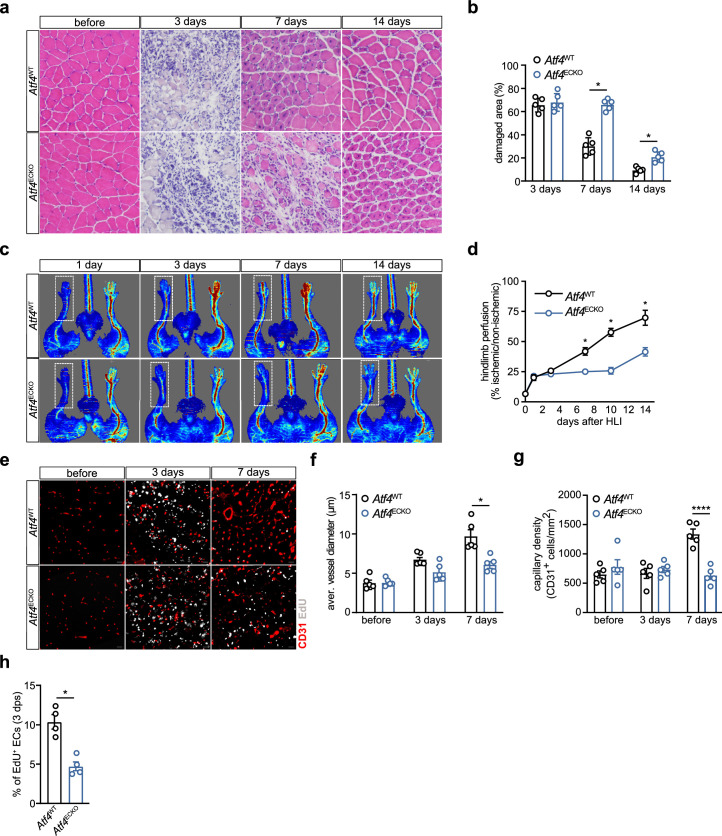
ATF4 is required in ECs for ischemia-induced revascularization in a preclinical model of PAD. **a**, Representative images of haematoxylin and eosin (H&E)-stained sections of gastrocnemius muscle from *Atf4*^WT^ (n = 5) and *Atf4*^ECKO^ (n = 5) mice. **b**, Quantification of percentage of regenerative area from panel **a** measured as areas with mononuclear cells infiltration and fibers with centrally localized nuclei. **c-d**, Representative laser Doppler images (**c**) and quantification of hindlimb perfusion (**d**) in *Atf4*^WT^ (n = 6) and *Atf4*^ECKO^ (n = 6) mice. **e**, Representative images of gastrocnemius muscle from *Atf4*^WT^ (n = 5) and *Atf4*^ECKO^ (n = 5) mice stained for the EC marker (CD31, red) and EdU (white) (Scale bar: 20 µm). **f-h**, Quantification of vessel diameter (**f**), vessel density (**g**) and percentage of proliferative (EdU^+^) ECs (**h**) from panel **e**. Each dot represents a single mouse in panels **b**, **f**, **g** and h or mean ± SEM in panel **d**. Two-way ANOVA with Sidak’s multiple comparisons (^*^p < 0.05) was used in **b**, **d**, **f** and **g**. Student’s t test (two-tailed, unpaired, parametric, ^*^p < 0.05) was used in **h**. P = 0.0007 (**d**, 7 days), P < 0.0001 (**d**, 10 days), P < 0.0001 (**d**, 14 days), P < 0.0001 (**f**, 7 days), P < 0.0001 (**g**, 7 days), P = 0.0019 (**h**). Source data

We next analyzed the functional contribution of *ATF3/ATF4*^+^ ECs in PAD by using a preclinical mouse model. We assessed muscle reperfusion after hindlimb ischemia (HLI) in wild-type and EC-specific inducible ATF4 knockout mice (*Atf4*^ECKO^). HLI efficiently reduced blood flow in both genotypes, leading to similar levels of ischemia-induced damage 3 days after surgery (Extended Data Fig. 3a,b), characterized by large degenerative areas with cell infiltration and regenerating fibers. *Atf4*^ECKO^ mice exhibited impaired blood flow recovery starting 7 days after HLI (Extended Data Fig. 3c,d), which coincided with reduced vessel diameter and density (Extended Data Fig. 3e–g) and lower EC proliferation (Extended Data Fig. 3h). As a result, *Atf4*^ECKO^ mice showed larger damaged muscles 7 days and 14 days after surgery (Extended Data Fig. 3a,b), illustrating the functional importance of *ATF3/ATF4*^+^ ECs to ischemia-induced revascularization in a preclinical model of PAD.

### ECs undergo profound transcriptomic rewiring during PAD

**Extended Data Fig. 4:**
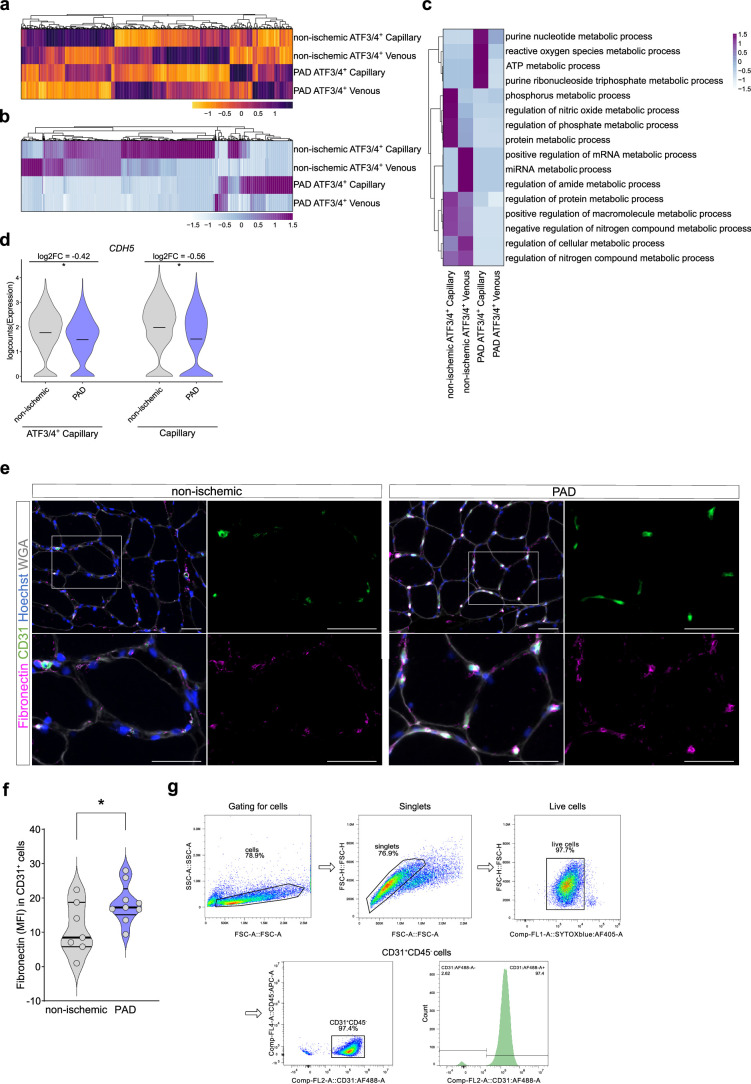
*ATF3/ATF4*^+^ ECs undergo a profound transcriptomic rewiring in PAD. **a**, Heatmap of centered logcounts of DEGs (adjusted p-value < 0.05) between *ATF3/ATF4*^+^ ECs in non-ischemic and PAD, color indicates the centered logcount value. **b-c**, Heatmap of centered values from ORA analysis over the DEGs in *ATF3/ATF4*^+^ Venous and Capillary Ecs between conditions, color indicates the centered values. Panel **b** displays all the enriched processes and panel **c** focuses on metabolic processes. **d**, Violin plots showing logcounts values of *CDH5* expression between conditions, a negative log2FC indicates a downregulation in PAD (^*^p < 0.05), horizontal line indicates the average logcounts value. **e**, Representative images of gastrocnemius muscle from non-ischemic (n = 7) and PAD (n = 9) patients stained for Fibronectin (magenta), the EC marker (CD31, green) cell nuclei (Hoechst, blue) and WGA (white) (Scale bar: 50 µm). In each group, the top left panel has a lower magnification compared to the other 3 pictures. White rectangle indicates the zoomed area displayed in the remaining panels **f**, Quantification of Fibronectin mean fluorescence intensity (MFI) in CD31^+^ cells from panel **e**. **g**, Flow cytometric analysis of percentage of CD31^+^CD45^−^ cells in primary cells isolated from human gastrocnemius muscle (related to Fig. 3, see Methods). Each dot represents a single patient in panel **f**. Wilcoxon Rank Sum test (as implemented in Seurat package) was used in **a** and **d**. Student’s t test (two-tailed, unpaired, parametric, ^*^p < 0.05) was used in **f**. P = 1.04e-19 (**d**, *ATF3/ATF4*^+^ Capillary), P = 6.11e-39 (**d**, Capillary), P = 0.0454 (**f**). Source data

Next, we assessed the transcriptional differences between conditions by calculating the number of differentially expressed genes (DEGs) that are specific for each EC population (Fig. 2h and Supplementary Data 1). *ATF3/ATF4*^+^ venous and *ATF3/ATF4*^+^ capillary ECs showed the highest number of DEGs between non-ischemic and PAD samples (Fig. 2i). ORA on the DEGs in *ATF3/ATF4*^+^ venous or *ATF3/ATF4*^+^ capillary ECs (Extended Data Fig. 4a and Supplementary Data 1) revealed that few enriched processes were activated in PAD (Extended Data Fig. 4b and Supplementary Data 1), whereas most of them were downregulated, indicating that many functions exerted by these populations are repressed, including angiogenesis, immune regulation, leukocyte interactions as well as metabolism (Fig. 2j and Extended Data Fig. 4c). Conversely, few gene sets related to epithelial-to-mesenchymal transition (EMT), platelet activation, wound healing and cellular responses to metal ions were activated in PAD (Fig. 2j).

The activation of EMT in *ATF3/ATF4*^+^ capillary ECs (Fig. 2j) prompted us to EndoMT. Focusing on capillary ECs, we noticed EndoMT features^32^, including repressed fatty acid metabolism genes and partial mesenchymal activation (Fig. 2k). Some ‘canonical’ EndoMT genes, such as *SNAI1*, *S100A4* and *SERPINE1*, were not affected in PAD, whereas several extracellular matrix (ECM) components, such as *COL1A1*, *COL1A2* and *FN1*, were highly upregulated (Fig. 2k). This phenotype was present in both capillary subpopulations, and they also lowered *CDH5* expression but did not completely lose it (Extended Data Fig. 4d), suggesting partial EndoMT. We then investigated how ECs rewire their matrisome—a set of genes that defines ECM composition and function^33^. Capillary ECs in PAD changed only 11.7% (32/274) of the core matrisome genes but showed significant activation of ECM components, such as collagens, *FN1* and *FBN1*, as well as alterations in components of the basement membrane, such as laminins, *HSPG2* and *NID2* (Fig. 2l). Immunohistological analysis confirmed increased expression of the mesenchymal marker fibronectin (FN1) by CD31^+^ ECs in PAD (Extended Data Fig. 4e,f). Taken together, ECs in PAD undergo profound transcriptomic alterations that impair angiogenesis and immunoregulatory functions but promote EndoMT.

**Fig. 3:**
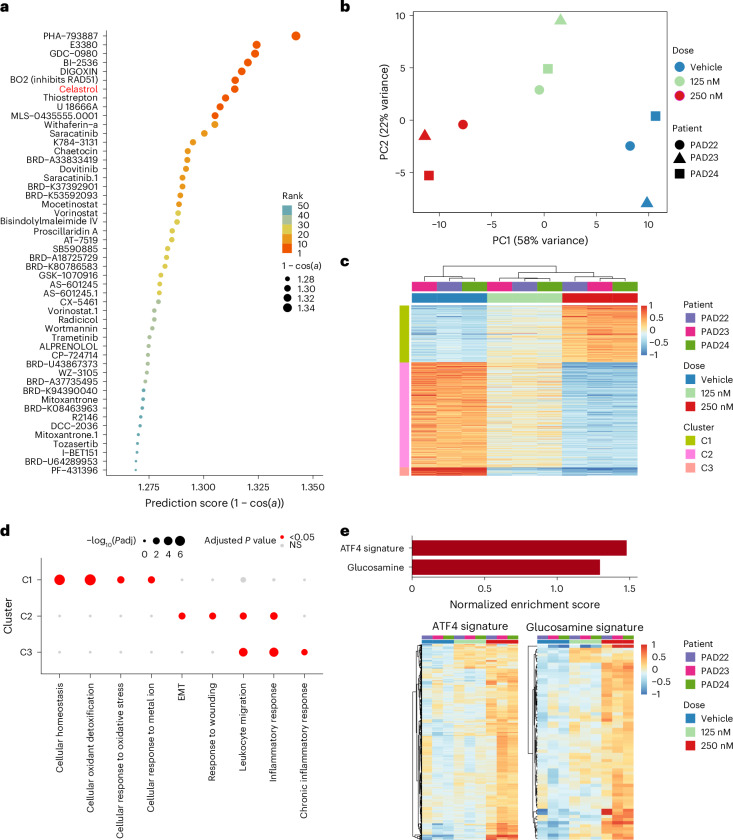
Celastrol partially reverses the EC PAD signature. **a**, Dot plot of prediction scores (derived from the L1000CDS^2^ search engine) from different compounds with the potential to reverse EC PAD signature. Color and size of the dots indicate the rank and the prediction score, respectively. **b**, Principal component analysis showing sample distances between different ECs (isolated from patients with PAD) treated with vehicle (DMSO) or celastrol (125 nM or 250 nM). Color and shape indicate treatment and patient of origin, respectively. **c**, Heatmap showing centered normalized values of DEGs (adjusted *P* < 0.05) between vehicle and celastrol 250 nM; color indicates the centered normalized value. Genes (rows) are organized and color coded based on clustering of gene expression profiles. **d**, ORA of the gene clusters from **c**; color and size indicate adjusted *P* values. **e**, Bar plot (top panel) showing normalized enrichment score (adjusted *P* < 0.05) from GSEA using ATF4 (Fan et al.^24^) and glucosamine (Alhusban et al.^39^) signatures. Heatmap (bottom panel) showing centered normalized values of top enriched genes from each signature; color indicates the centered normalized value. Wald test (as implemented in the DESeq2 package) was used in **c**. Fisher’s one-tailed test (as implemented in g:Profiler) was used in **d**. Adaptive multilevel Monte Carlo scheme (as implemented in the fgsea package) was used in **e**. PC, principal component.

Finally, we used DEGs between PAD and non-ischemic ECs to predict drugs^34^ that can reverse the PAD EC phenotype. This analysis predicted a set of compounds with this therapeutic potential (Fig. 3a), including withaferin A or saracatinib, drugs that target inflammation and endothelial dysfunction^35,36^. We focused on celastrol for its ability to improve vascular remodeling and to reduce endothelial dysfunction^37^. We treated primary isolated muscle ECs (Extended Data Fig. 4g and Methods) from three patients with PAD for 24 hours with vehicle (DMSO) or celastrol (125 nM or 250 nM (ref. ^38^)). RNA-seq analysis showed a dose-dependent effect across patients (Fig. 3b,c and Supplementary Data 1), including upregulated (C1 in Fig. 3c) and downregulated (C2 and C3) genes. ORA (Fig. 3d) confirmed celastrol-dependent upregulation of processes related to cellular homeostasis, detoxification and response to metal ions (C1) while downregulating EMT, response to wounding (C2), leukocyte migration and inflammation (C2 and C3), indicating the ability of celastrol to partially reverse the PAD phenotype observed in the scRNA-seq dataset (Fig. 2j). Celastrol also activated ATF4 or glucosamine signatures (Fig. 3e), which improves perfusion recovery in PAD preclinical models by activating ATF4 (ref. ^39^). These findings suggest the potential of our dataset for identifying compounds that could potentially revert PAD phenotypes.

### *LYVE1*^hi^MHCII^low^ macrophages are the dominant macrophage population in PAD

**Fig. 4:**
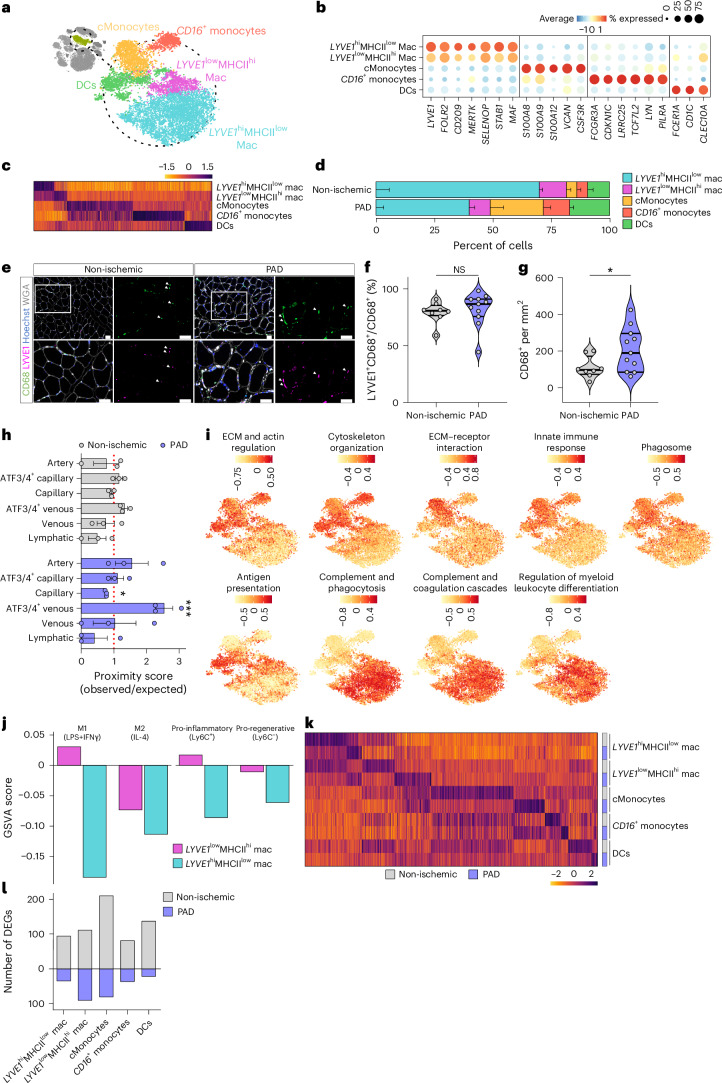
*LYVE1*^hi^MHCII^low^ macrophages are the dominant macrophage population in PAD. **a**, t-SNE plot of MoMac subtypes identified from human gastrocnemius muscle, color coded by the identified subtypes. **b**, Dot plot of centered log count values from MoMac subtype-specific marker genes. Dot color indicates centered log counts, and size reflects the proportion of cells expressing the gene. **c**, Heatmap of centered log counts of DEGs (adjusted *P* < 0.05) between MoMac subtypes; color indicates the centered log count value. **d**, Stacked bar plots showing cluster percentage from non-ischemic patients (*n* = 4) and patients with PAD (*n* = 4), color coded by cluster. Each stack shows mean − s.e.m. **e**, Representative images of gastrocnemius muscle from non-ischemic (*n* = 8) and PAD (*n* = 11) stained for a pan-macrophage marker (CD68, green), LYVE1 (magenta), cell nuclei (Hoechst, blue) and WGA (white). Top left panel has a lower magnification compared to the other three pictures. White rectangle indicates the zoomed area displayed in the remaining panels. Scale bars, 50 µm. **f**,**g**, Quantification of the percentage of LYVE1^+^CD68^+^ cells from the total of CD68^+^ cells (**f**) and the total number of macrophages measured as the number of CD68^+^ cells per mm^2^ (**g**). **h**, Bar plot showing proximity scores (Methods) of *LYVE1*^hi^MHCII^low^ macrophages to each EC subtype in the spatial dataset from non-ischemic patients (*n* = 3) and patients with PAD (*n* = 3), color coded by condition. Each bar represents mean ± s.e.m. **i**, t-SNE plots showing GSVA scores for different macrophage activation processes as defined by Sanin et al.^48^, color coded by GSVA score. **j**, GSVA scores from different pro-inflammatory and pro-regenerative signatures as defined by Varga et al.^50^ between *LYVE1*^hi^ and *LYVE*^low^ macrophages, color coded by cluster. **k**, Heatmap of centered log counts of DEGs (adjusted *P* < 0.05) specific for each MoMac subtype in each condition; color indicates the centered log count value. **l**, Bar plot showing the number of DEGs for each MoMac subtype in each condition; color indicates the condition. Each dot represents a single patient in **f**–**h**. Wilcoxon rank-sum test (as implemented in the Seurat package) was used in **c** and **k**. Student’s *t*-test (two-tailed, unpaired, parametric, NS > 0.05) was used in **f**, and Mann–Whitney *U*-test (two-tailed, unpaired, non-parametric, *P < 0.05) was used in **g**. Binomial test (one-tailed, observed versus expected, *P < 0.05) was used in **h**. *P* = 0.3511 (**f**), *P* = 0.0473 (**g**), *P* = 0.0066 (**h**, PAD capillary), *P* = 0.0007 (**h**, PAD *ATF3/ATF4*^+^ venous). DC, dendritic cell; Mac, macrophage. Source data

**Extended Data Fig. 5:**
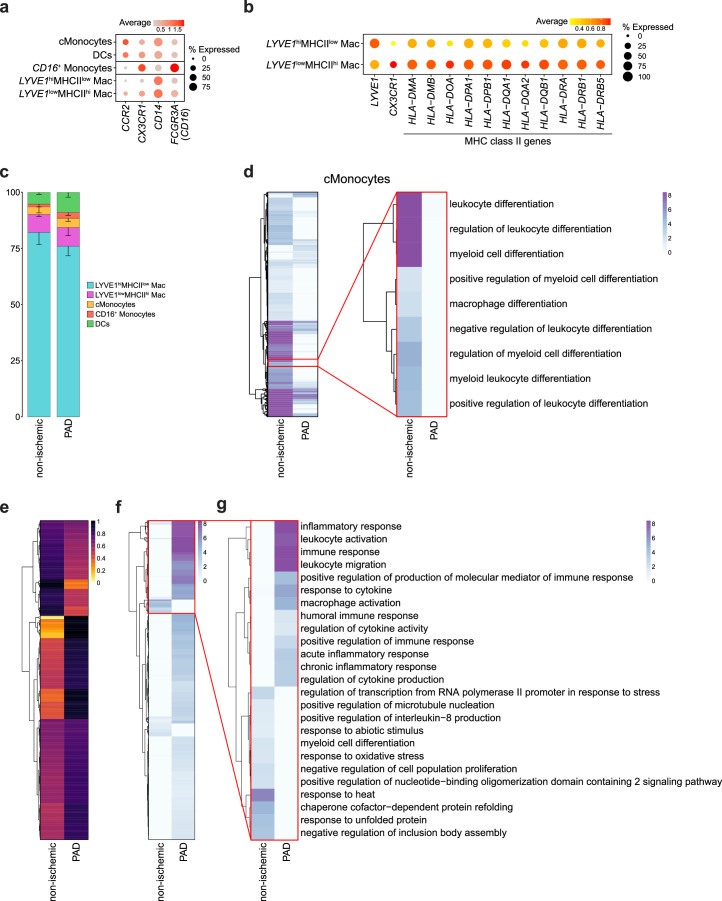
*LYVE1*^hi^MHCII^low^ macrophages get activated in PAD. **a**, Dot plot of logcount values of markers from different myeloid populations in Monocytes/Macrophages subtypes. Color and size of the dots indicate the logcount value and the proportion of cells that express the gene, respectively. **b**, Dot plots of logcounts values of *LYVE1*, *CX3CR1* and MHC-II class genes in *LYVE1*^hi^MHCII^low^ and *LYVE1*^low^MHCII^hi^ macrophages. Color and size of the dots indicate the logcount value and the proportion of cells that express the gene, respectively. **c**, Stacked bar plots showing cluster percentage in each condition in the spatial dataset from non-ischemic (n = 3) and PAD (n = 3) patients, color-coded by cluster. Each stack represents mean - SEM. **d**, Heatmap of centered values from ORA analysis over the DEGs in cMonocytes between conditions, color indicates the centered values. The left heatmap shows all identified enriched processes, while the right heatmap only shows terms related to leukocyte/myeloid cell/macrophage differentiation. **e**, Heatmap of logcounts values of DEGs (adjusted p-value < 0.05) between *LYVE1*^hi^MHCII^low^ macrophages in non-ischemic and PAD, color indicates the logcount value. **f-g**, Heatmap of centered values from ORA analysis over the DEGs in *LYVE1*^hi^MHCII^low^ macrophages between conditions, color indicates the centered values. Panel **f** displays all the enriched processes and panel **g** focuses on top enriched processes in each condition. Wilcoxon Rank Sum test (as implemented in Seurat package) was used in **e**. Source data

In experimental PAD models, monocyte-derived macrophages contribute to vascularization^16,19,21,40–42^. However, acute mouse models do not reflect chronic scenarios, so the contribution of macrophages to human PAD is poorly understood. We decided to further investigate the MoMac cluster, selecting these cells from the complete dataset where we identified and annotated five distinct populations^43,44^ (Fig. 4a–c and Supplementary Data 1). This dataset included classical monocytes (cMonocytes), *CD16*(*FCGR3A*)^+^ monocytes, dendritic cells and two macrophage populations. Alternatively, we could also classify the populations as *CCR2*^+^*CX3CR1*^low^*CD14*^hi^*CD16*^low^ (cMonocytes and dendritic cells) and *CCR2*^−^*CX3CR1*^hi^*CD14*^low^*CD16*^hi^ (*CD16*^+^ monocytes) (Extended Data Fig. 5a).

Macrophage subpopulations were annotated as *LYVE1*^hi^MHCII^low^*CX3CR1*^low^ and *LYVE1*^low^MHCII^hi^*CX3CR1*^hi^ (Extended Data Fig. 5b), matching previous characterization^45^. In mouse, *LYVE1*^hi^MHCII^low^ macrophages often have a perivascular location and a distinct ontology, phenotype and function compared to monocyte-derived macrophages^45^. *LYVE1*^hi^MHCII^low^ macrophages in our dataset expressed high levels of tissue-resident macrophage markers, including *LYVE1*, *FOLR2*, *CD209*, *MERTK*, *SELENOP*, *STAB1* and *MAF*^44,46,47^ (Fig. 4b). *LYVE1*^hi^ macrophages were the largest macrophage population, even though their percentage was lower in PAD (70 ± 6% and 40 ± 3%, respectively; Fig. 4d). LYVE1/CD68 co-staining confirmed the predominance of *LYVE1*^hi^ macrophages in both conditions (Fig. 4e,f), despite a significant increase in CD68^+^ MoMacs in PAD muscle (Fig. 4g), consistent with previous work^21^. The spatial dataset also showed *LYVE1*^hi^ macrophages as the main MoMac population in human muscle (Extended Data Fig. 5c). Notably, the percentage of *LYVE1*^+^ macrophages over the total fraction of MoMac cells (CD68^+^) was similar in both spatial and immunofluorescence approaches (≈80–85%; Fig. 4f and Extended Data Fig. 5c) and was not affected in PAD in none of them. Because the scRNA-seq dataset could include (due to residual blood in the sample) circulating MoMac populations such as cMonocytes and dendritic cells, the proportional reduction in *LYVE1*^+^ macrophages was likely secondary to increased presence of those populations in PAD (Fig. 4d). Moreover, the increase of cMonocytes (Fig. 4d), but not monocyte-derived *LYVE1*^low^MHCII^hi^ macrophages, suggests impaired monocyte-to-macrophage differentiation in PAD, which was confirmed using ORA (Extended Data Fig. 5d), or could be secondary to increased cMonocyte numbers (Fig. 4d).

To evaluate whether perivascular *LYVE1*^hi^ macrophages^45^ preferentially localize to specific vessels, we calculated a proximity score using spatial transcriptomics, defined as the ratio of observed versus expected *LYVE1*^hi^MHCII^low^ macrophages closest to a given EC subtype. A score higher than 1 indicates that *LYVE1*^hi^MHCII^low^ macrophages are usually closer to a specific EC subtype than expected by chance. In non-ischemic samples, we did not observe any spatial enrichment of *LYVE1*^hi^MHCII^low^ macrophages (Fig. 4h). However, in PAD, they exhibited a significant proximity score to *ATF3/ATF4*^+^ venous (Fig. 4h), indicating a preferential localization to these ECs.

MoMacs exert many functions depending on the biological context. Sanin et al.^48^ categorized macrophage activation into four functional pathways: phagocytic, oxidative stress, inflammatory and remodeling (regenerative). We used this framework to characterize the functional differences between the MoMac populations in our dataset. *CD16*^+^ monocytes, cMonocytes and dendritic cells were characterized by processes related to early-stage activation (ECM/cytoskeleton regulation) and inflammatory/oxidative paths (innate immune response and phagosome) (Fig. 4i). *LYVE1*^low^ macrophages and dendritic cells showed a profile of antigen presentation, consistent with their higher MHCII levels (Extended Data Fig. 5b) and with their previous characterization^45^. Both *LYVE1*^hi^ and *LYVE1*^low^ macrophages were characterized by a specific phagocytic profile as they were enriched in processes related to complement signaling, phagocytosis and regulation of leukocyte differentiation.

The differential and overlapping functions of *LYVE1*^hi^ versus *LYVE1*^low^ macrophages upon loss of muscle homeostasis, including PAD, are poorly understood, so we explored whether they present pro-inflammatory (M1) or anti-inflammatory, pro-regenerative (M2)^49^ macrophage signatures. We used published gene sets associated with M1 or M2 macrophages^50^ under in vitro stimulation as well as in vivo states of macrophages isolated from cardiotoxin-injured muscles (Ly6C^+^ macrophages at day 2 after injury (pro-inflammatory) and Ly6C^−^ at day 4 after injury (pro-regenerative)). *LYVE1*^hi^ macrophages expressed lower levels of both M1/inflammatory and M2/regenerative signatures (Fig. 4j), consistent with their proposed ‘homeostatic’ function. Additionally, *LYVE1*^low^ macrophages showed higher expression of both signatures, although they exhibited a more pronounced pro-inflammatory signature (Fig. 4j). Thus, *LYVE1*^hi^ macrophages do not reflect the genetic fingerprint of M1/inflammatory or M2/regenerative macrophages.

### *LYVE1*^hi^MHCII^low^ macrophages get activated during PAD

To assess whether MoMac populations are altered upon PAD, we detected DEGs for every individual population and observed alterations across all MoMac populations (Fig. 4k,l and Supplementary Data 1). Although cMonocytes showed the highest number of DEGs, most of their biological processes were downregulated in PAD, including terms related to leukocyte/macrophage differentiation (Extended Data Fig. 5d). Because *LYVE1*^hi^MHCII^low^ macrophages are the dominant population, and their role in muscle is poorly understood, we focused on those. Most DEGs in *LYVE1*^hi^MHCII^low^ macrophages were upregulated during PAD (Extended Data Fig. 5e and Supplementary Data 1) and associated with inflammatory and immune responses, cytokine production and macrophage activation (Extended Data Fig. 5f,g and Supplementary Data 1). The top repressed processes in PAD were related to heat responses and protein folding (Extended Data Fig. 5g), including genes such as *HSPA1A* and *HSPA1B*, which have been associated with atheroprotective and anti-inflammatory roles in macrophages^51,52^. Altogether, *LYVE1*^hi^MHCII^low^ macrophages get activated and acquire pro-inflammatory properties during PAD.

### Disruption of cellular communication networks in the muscle microenvironment during PAD

**Fig. 5:**
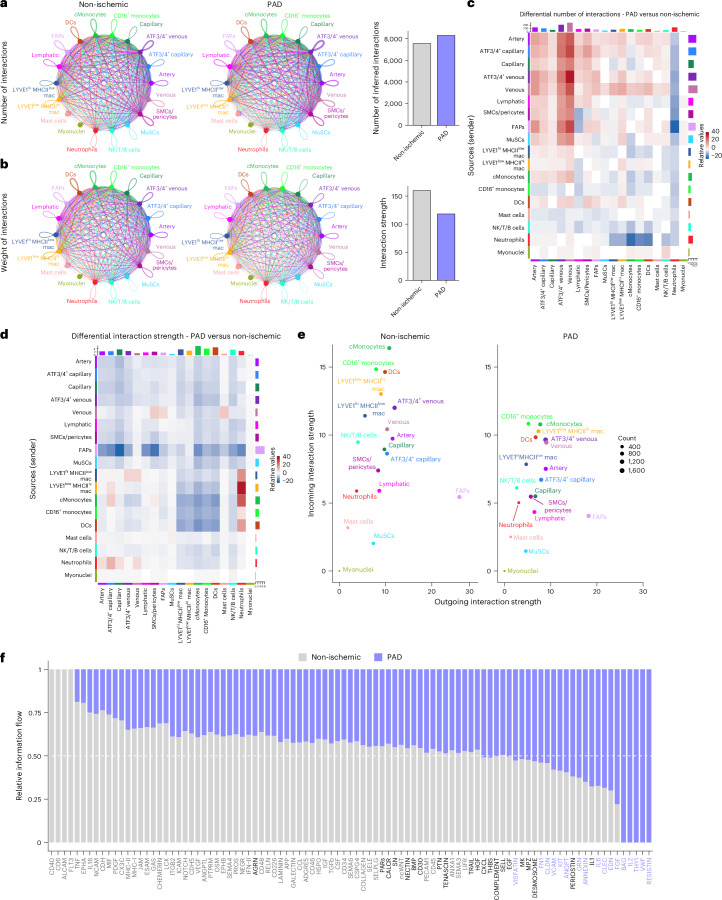
Cellular communication in human skeletal muscle is disrupted in PAD. **a**,**b**, Circle plots showing the number (**a**) and weight (**b**) of inferred interactions between cell types in non-ischemic and PAD (left and middle plots) and bar plots quantifying these parameters (right plots). Color and width of the edges indicate the sender and number or weight of interactions, respectively. **c**,**d**, Heatmaps showing increased (red) or decreased (blue) number (**c**) and weight (**d**) of inferred interactions in PAD. Labels on the left side indicate the cell type expressing the ligands and bottom labels the cell types receiving them. **e**, Scatter plots showing the strength of the outgoing (*x* axis) and incoming (*y* axis) interactions for each cell type in non-ischemic (left) and PAD (right). Color and size of the dots indicate the cell type and the number of inferred interactions, respectively. **f**, Bar plots showing the relative information flow for significant communication pathways between non-ischemic and PAD.

**Extended Data Fig. 6:**
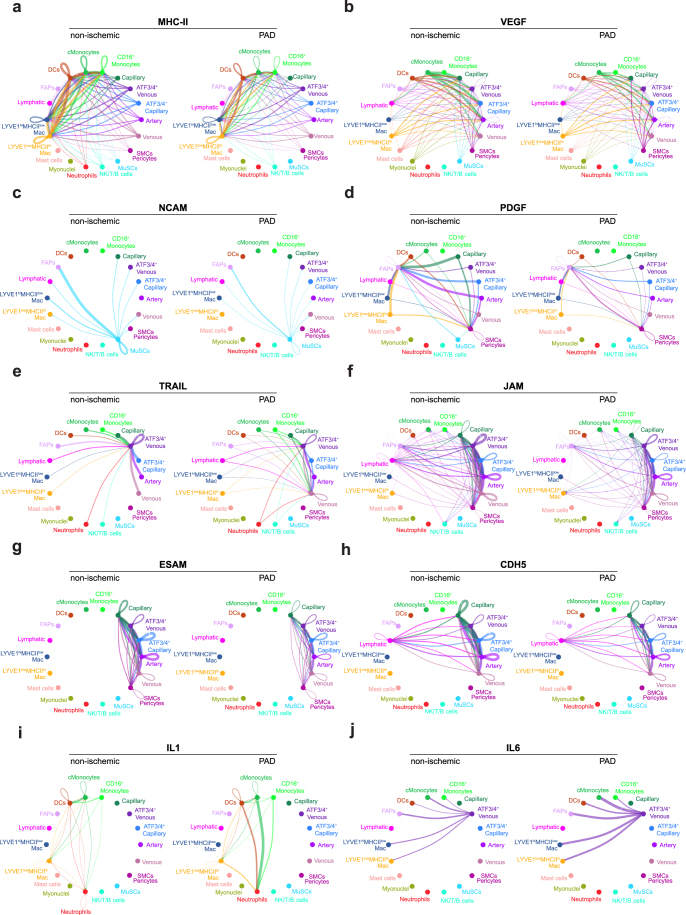
Cellular communication in human skeletal muscle is disrupted in PAD. **a-j**, Circle plots showing MHC-II (**a**), VEGF (**b**), NCAM (**c**), PDGF (**d**), TRAIL (**e**), JAM (**f**), ESAM (**g**), CDH5 (**h**), IL1 (**i**) and IL6 (**j**) communication in non-ischemic (left) and PAD (right). Color and width of the edges indicates the sender and weight of interactions, respectively.

To study cell–cell communication, we applied CellChat^53^ to the complete scRNA-seq dataset. During PAD, the number of interactions slightly increased (Fig. 5a), although the strength of these interactions was generally reduced (Fig. 5b). The increased number of interactions was mainly mediated by ECs, SMCs/pericytes, FAPs and MuSCs (Fig. 5c). Only neutrophils showed an increase in interaction strength with MoMac populations during PAD (Fig. 5d). FAPs were the main signal senders in the non-ischemic muscle microenvironment, whereas MoMac populations were the main receptors (Fig. 5e). In PAD, most populations reduced their interaction strength (Fig. 5e), suggesting disrupted communication in the PAD muscle microenvironment. Indeed, most pathways were reduced in PAD (Fig. 5f). MHCII signaling was one of the top reduced communications (Fig. 5f), with MoMacs and ECs as the main cell types involved (Extended Data Fig. 6a). ECs reduced VEGF signaling in PAD (Extended Data Fig. 6b). MuSCs showed reduced NCAM-mediated interactions with FAPs (Extended Data Fig. 6c). ECs and *LYVE1*^+^ macrophages reduced PDGF signaling with FAPs (Extended Data Fig. 6d), a pathway involved in muscle hypertrophy and regeneration^54^. *ATF3/ATF4*^+^ venous showed reduced TRAIL signaling (Extended Data Fig. 6e), a key mediator in generating stable vessels in preclinical PAD models^55^. ECs also reduced interactions of several tight and adherens junctional genes, including JAM, ESAM and CDH5 (Extended Data Fig. 6f–h). On the other hand, pro-inflammatory IL-1 and IL-6 were enriched in PAD, with the latter being specifically produced by *ATF3/ATF4*^+^ venous ECs (Extended Data Fig. 6i,j). All detected interactions and ligand–receptor pairs are available in Supplementary Data 1.

### Altered SMC/pericyte–endothelium interactions suggest impaired vessel homeostasis during PAD

**Fig. 6:**
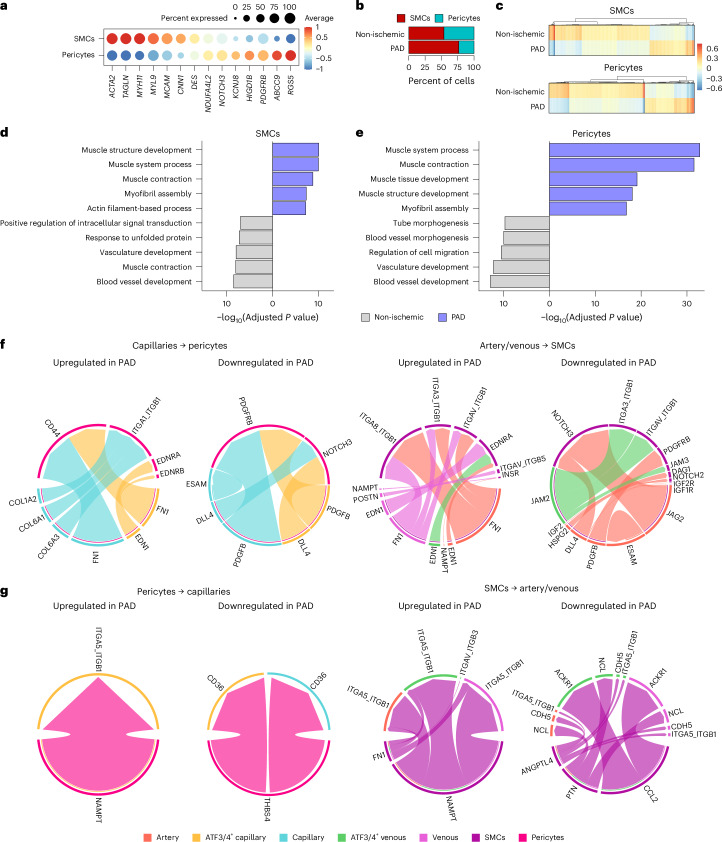
Cellular communication between mural cells and ECs indicates reduced vessel homeostasis. **a**, Dot plot of centered log count values from SMC and pericyte marker genes. Color and size of the dots indicate the centered log count values and the proportion of cells that express the gene, respectively. **b**, Stacked bar plots showing cluster percentage in each condition, color coded by cluster. Each stack represents mean − s.e.m. **c**, Heatmap of centered log counts of DEGs (adjusted *P* < 0.05) from SMCs (top) and pericytes (bottom) between conditions; color indicates the centered log count value. **d**,**e**, ORA of top biological processes of DEGs from **c** in SMCs (**d**) and pericytes (**e**); color indicates in which condition the process is significant (adjusted *P* < 0.05). **f**,**g**, Circle plots of ligands and receptors upregulated/downregulated in PAD from ECs to mural cells (**f**) and from mural cells to ECs (**g**). Color and width of the edges indicate the sender and weight of interactions, respectively. Wilcoxon rank-sum test (as implemented in the Seurat package) was used in **c**. Fisher’s one-tailed test (as implemented in g:Profiler) was used in **d** and **e**.

SMCs/pericytes are essential for proper vessel function and stability. We, therefore, investigated how they interact with ECs during PAD. First, we annotated them based on well-known marker genes of both populations^56^ (Fig. 6a). Although they were present in similar proportions in non-ischemic samples (Fig. 6b), SMCs comprised approximately 75% of mural cells in PAD. Most DEGs were downregulated during PAD (Fig. 6c and Supplementary Data 1), but they increased pathways related to muscle development and contractility (Fig. 6d,e) while downregulating blood vessel and vasculature development processes.

CellChat to study capillary–pericyte and artery/venous–SMC interactions revealed that ECs mainly increased FN1 signaling to both pericyte and SMC populations (Fig. 6f), whereas capillaries and arteries reduced PDGFB–PDGFRB and JAG2/DLL4–NOTCH3 signaling, key mediators of recruitment and attachment to the endothelium^57,58^. Conversely, SMCs/pericytes increased NAMPT signaling to ECs during PAD (Fig. 6g) but reduced THBS4 (pericytes) and *PTN*, *CCL2* and *ANGPTL4* (SMCs) communication (Fig. 6g). Together, these changes suggest a reduced capacity of SMCs/pericytes to maintain vessel homeostasis and endothelial attachment in PAD.

### EC–macrophage crosstalk alters signaling networks driving inflammation and immune modulation in *LYVE*^hi^MHCII^low^ macrophages during PAD

**Extended Data Fig. 7:**
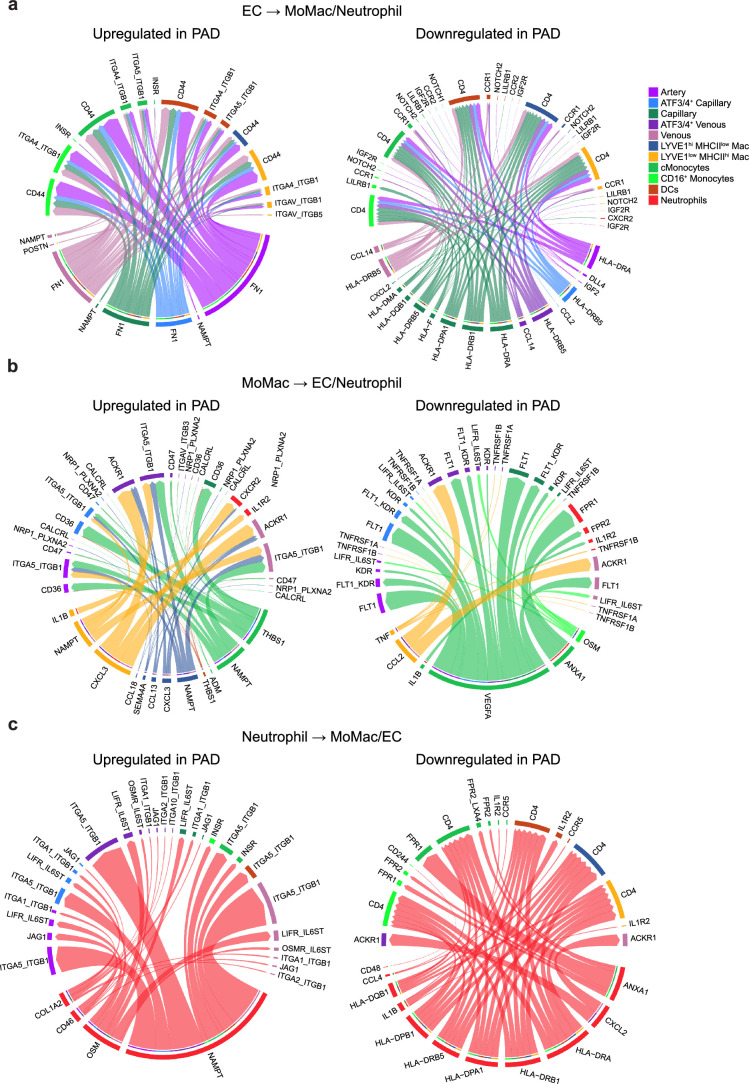
Reciprocal communication between EC-MoMac-Neutrophil promotes pro-inflammatory and anti-angiogenic communication during PAD. **a-c**, Circle plots showing upregulated (left panel) or downregulated (right panel) communication during PAD from ECs (**a**), MoMacs (**b**) and Neutrophils (**c**) to the other cell types. Color and width of the edges indicates the sender and weight of interactions, respectively.

Finally, we analyzed EC–MoMac reciprocal communication, including neutrophils due to their increased interaction strength with MoMacs during PAD (Fig. 5d). ECs upregulated FN1 interactions with all MoMacs while reducing MHCII signaling (Extended Data Fig. 7a). MoMacs increased *NAMPT*, linked to pro-inflammatory markers in patients with CAD^59^, and *THBS1* (cMonocyte-specific) signaling, elevated in patients with PAD and with anti-angiogenic effects in ECs^60^. Conversely, they downregulated *VEGFA* and *ANXA1* signaling (important for inflammatory resolution in muscle^61^) with ECs and neutrophils, respectively (Extended Data Fig. 7b). Neutrophils upregulated *NAMPT* and *OSM*, a cytokine promoting EC inflammation and neutrophil recruitment^62,63^, while downregulating MHCII genes and *ANXA1*, further suggesting impaired inflammatory resolution (Extended Data Fig. 7c).

**Fig. 7:**
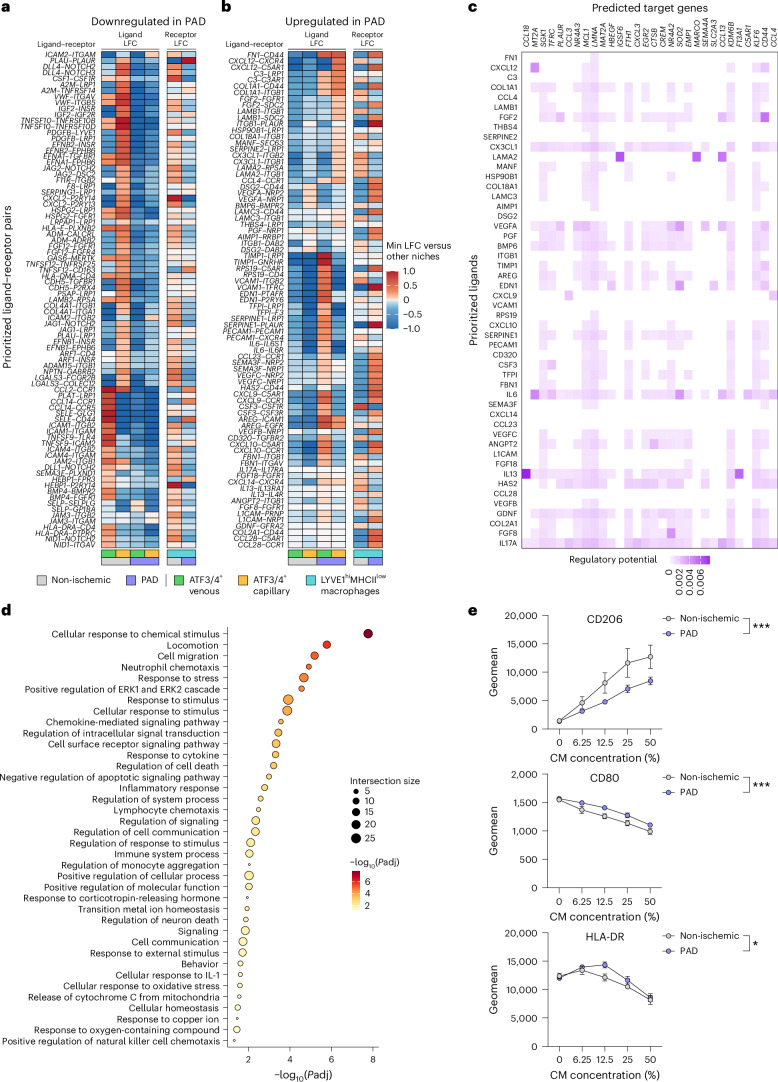
Cellular communication between *ATF3/ATF4*^+^ ECs and *LYVE1*^hi^MHCII^low^ macrophages is altered during PAD. **a**,**b**, Heatmaps showing downregulated (**a**) and upregulated (**b**) ligand–receptor pairs in PAD. In each panel, the left part indicates the ligand log fold change (LFC) in *ATF3/ATF4*^+^ ECs and the right part the receptor LFC in *LYVE1*^hi^MHCII^low^ macrophages from each ligand–receptor pair. Color indicates the LFC. **c**, Heatmap showing the regulatory potential of *ATF3/ATF4*^+^ ECs ligands upregulated in PAD (left labels) over the downstream target genes upregulated in *LYVE1*^hi^MHCII^low^ macrophages (top labels). Color indicates the regulatory potential. **d**, Dot plot showing ORA results from the predicted targets in **c**. Color and size of the dots indicate −log_10_(adjusted *P* value) and number of genes in each category (intersection size), respectively. **e**, Graphs showing the geomean expression ± s.e.m. of the markers CD206, CD80 and HLA-DR on human CD14^+^ cells cultured in vitro with increasing concentrations of EC-derived conditioned media (CM)—non-ischemic CM (gray, *n* = 3) and PAD CM (blue, *n* = 5). Fisher’s one-tailed test (as implemented in g:Profiler) was used in **d**. Two-way ANOVA (*P < 0.05, ***P < 0.001) was used in **e**. *P* = 0.0002 (**e**, CD206), *P* = 0.0001 (**e**, CD80), *P* = 0.0414 (**e**, HLA-DR). Min, minimum. Source data

Next, we applied NicheNet^64^, which can additionally link ligand–receptor pairs to their downstream signaling networks, to further study EC–macrophage interactions in PAD. Because *ATF3/ATF4*^+^ ECs present an immunomodulatory profile, and *ATF3/ATF4*^+^ venous were the closest to *LYVE1*^hi^MHCII^low^ macrophages in PAD (Fig. 4h), we focused on the communication between those populations. NicheNet identified different ligand–receptor pairs specifically downregulated (Fig. 7a) or upregulated (Fig. 7b) in PAD samples, indicating a profound rewiring of the communication network between *ATF3/ATF4*^+^ ECs and *LYVE1*^hi^MHCII^low^ macrophages. Some of the top reduced signals during PAD were related to *GAS6* and *CSF1* signaling (Fig. 7a), whereas *IL6* signaling was enriched (Fig. 7b). We also used NicheNet to detect which upregulated genes in *LYVE1*^hi^MHCII^low^ macrophages during PAD could be downstream targets of ligands produced by *ATF3/ATF4*^+^ ECs (Fig. 7c). These target genes included *CCL18*, *SGK1*, *PLAUR*, *CCL3*, *LMNA*, *HBEGF*, *IGFS6*, *FTH1* and *CTSB* (Fig. 7c) and are associated with migration, cytokine response, inflammatory response and immune-related processes (Fig. 7d).

To explore the ability of ECs to influence in vitro monocyte-to-macrophage differentiation in PAD, we generated conditioned media from primary muscle ECs from non-ischemic patients (*n* = 3) and patients with PAD (*n* = 5) and exposed it to CD14^+^ blood cells from healthy donors (*n* = 6). Fluorescence-activated cell sorting (FACS) analysis showed that PAD-derived conditioned media reduced CD206 expression (M2/regenerative) while increasing CD80 and HLA-DR (M1/inflammatory) in CD14^+^ cells (Fig. 7e), indicating that PAD EC-derived factors skew CD14^+^ cells toward a pro-inflammatory profile. Although these experiments used blood CD14^+^ cells rather than specific subtypes from our scRNA-seq dataset, they provide supportive evidence that altered crosstalk in muscle ECs during PAD could influence immune cell behavior.

## Discussion

We generated a comprehensive scRNA-seq dataset with over 100,000 cells from the muscle microenvironment of patients with PAD as well as non-ischemic comorbidity-matched controls (undergoing bypass surgery for an aneurysm). Pseudobulk analysis revealed increased TNF and IL-6 signaling, both associated with walking impairments in patients with PAD^65,66^. Other upregulated processes such as inflammation, unfolded protein response and metabolism were also reported in previously bulk RNA-seq studies of PAD muscle^67,68^. In contrast to our approach, those bulk studies are dominated by myonuclear transcripts, suggesting common pathway alterations across cell types. Our dataset provides a resource for cell-type-specific analysis of transcriptomics changes in PAD. Although here we focused on specific cell types, other cell (sub)populations may also critically contribute to PAD progression.

Besides identifying common EC subpopulations, we also found *ATF3/ATF4*^+^ venous and capillary ECs in human skeletal muscle, confirming earlier work in mice which showed that ATF3/ATF4^+^ ECs are functionally required for exercise-induced angiogenesis^24^. A venous population, transcriptionally equivalent to *ATF3/ATF4*^+^ venous, is specifically enriched in human skeletal muscle, adipose tissue and lymph nodes^69^. Also, glucosamine-driven stimulation of ATF4 signaling improves perfusion recovery in preclinical models of PAD^39^. Using this preclinical model, we showed that ATF4 deletion in ECs prevents ischemia recovery, further underscoring the critical contribution of ATF4 in ECs. Our scRNA-seq and spatial data revealed fewer *ATF3/ATF4*^+^ ECs in PAD while also reducing their angiogenic and immune regulatory pathways. ECs in PAD upregulated genes related to EndoMT and cellular responses to metal ions. Elevated levels of these ions have been described in patients with PAD and atherosclerotic lesions^70–73^ and have been linked to endothelial dysfunction^74,75^. These results suggest that ECs undergo maladaptive adaptations that impair their functions in PAD. Through in silico drug repurposing, we identified celastrol as a promising candidate to reprogram PAD ECs toward a healthier phenotype, which highlights the potential of our scRNA-seq data for therapeutic discovery.

Blood vessel formation and remodeling is heavily influenced by resident and recruited cells. In experimental models of PAD, monocyte-derived macrophages play a role in vascularization^16,19,21,40–42^ and muscle repair. After acute muscle ischemia or injury, macrophages adopt a pro-inflammatory/M1-like phenotype. Rapidly thereafter, M1 macrophages repolarize to an anti-inflammatory (regenerative)/M2-like phenotype^76,77^, which is required for optimal muscle regeneration^77–79^. M2-like macrophages also have an important pro-angiogenic role because they are a source of VEGF. Nonetheless, acute mouse models differ from chronic PAD, so the contribution of macrophages in PAD remains unclear. We measured an almost two-fold increase in CD68^+^ cells, predominantly *LYVE1*^hi^ macrophages and not monocyte-derived *LYVE1*^low^ macrophages. This observation is interesting because preclinical mouse models for muscle ischemia have unequivocally supported the requirement of monocyte-derived macrophages for muscle regeneration^80,81^. It is unclear whether the failed accumulation of these cells in patients with PAD is a result of the chronic nature of PAD or contribute to the impaired regenerative response.

*LYVE1*^hi^MHCII^low^ macrophages were the dominant muscle macrophage population, even under a setting of chronic inflammation such as PAD. These cells were previously identified as embryonically derived, locally self-renewing tissue-resident macrophages^44,82^. They cloak microlesions to prevent widespread neutrophil recruitment^83^ or clear damage-induced apoptotic cells^47^, but their contribution to PAD muscle pathology is not known. Consistent with mouse literature, *LYVE1*^hi^MHCII^low^ macrophages in our human muscle samples expressed tissue-resident markers including *FOLR2* and other M2/regenerative marker genes such as *CD163* and *MRC1* (CD206). However, during PAD, *LYVE1*^hi^MHCII^low^ macrophages also activated a pro-inflammatory gene program. The co-expression of genes that are typically associated with an M1-like as well as an M2-like state might explain the contrasting findings reported in literature. Recently, TIMD4 was proposed as a marker for resident mouse muscle macrophages, but we could not detect *TIMD4* expression in human macrophages. Also, we found that *LYVE1*^hi^MHCII^low^ macrophages preferentially localize close to *ATF3/ATF4*^+^ ECs in PAD and showed altered (more pro-inflammatory) crosstalk with ECs. Whether this altered EC–macrophage communication drives PAD muscle pathology or reflects a failed homeostatic response still needs further research.

Analysis of the transcriptomic changes in microvascular capillary ECs revealed partial EndoMT in PAD. EndoMT has been observed in patients with atherosclerosis^84^ and in patients with PAD^5^, and ischemia promotes the activation of EndoMT in several pathological settings^85^. Also, partial EndoMT is required for optimal angiogenesis in myocardial infarction^32^. It is possible that persistent hypoxia contributed to a partial and chronic EndoMT. Although we did observe activation of some, but not all, markers of EndoMT in PAD capillary ECs, ECM genes were significantly activated. Also, the endothelial marker *CDH5* was reduced, but not lost, in PAD. Interestingly, this state of partial EndoMT might be reversible^32^.

We detected strong cellular crosstalk between different cell types that was significantly altered in PAD, with downregulated signaling networks related to muscle regeneration, angiogenesis and vessel junctions and upregulated pro-inflammatory pathways such as *IL1B* or *IL6*. IL-1B is a known regulator of EndoMT^86^, and IL-1B inhibitors improved maximum and pain-free walking distance in patients with symptomatic PAD^87^, although muscle pathology was not further investigated in this study. Analyzing SMC/pericyte–EC interactions revealed a reduction in *PGDFRB* and *NOTCH3* signaling in PAD, which control mural cell recruitment and attachment to the endothelium. Also, mural cells increased *NAMPT* signaling, which is associated with pro-inflammatory markers in patients with coronary artery disease^59^. Both SMCs and pericytes upregulated contractility genes such as *ACTA2*, *TAGLN*, *MYH11* and *CNN1*, in line with increased contraction of vessels by pericytes under ischemia^88,89^. This phenotype might contribute to reduced perfusion in pathologies such as PAD^90^. Finally, we found that *LYVE1*^hi^MHCII^low^ macrophage activation could be EC dependent. During PAD, ECs reduced *CSF1* and *GAS6* signaling with *LYVE1*^hi^MHCII^low^ macrophages. CSF1 is the primary growth factor responsible for macrophage differentiation and survival^91^, and endothelial CSF1 contributes to this^92^. Inhibition of GAS6 signaling in macrophages increases their inflammatory responses^93^, which would be consistent with the activated state of *LYVE1*^hi^MHCII^low^ macrophages and their increased cytokine production (Extended Data Fig. 5g). The EC ligands upregulated in PAD furthermore seem to control downstream genes in *LYVE1*^hi^MHCII^low^ macrophages related to inflammation, migration and cytokine response. We experimentally showed that secreted factors from ECs during PAD increased the expression of M1 markers while reducing M2 markers in human CD14^+^ immune cells, further supporting a more pro-inflammatory profile of ECs during PAD. Beyond having a perivascular localization, these data imply that ECs might control the fate of *LYVE1*^hi^MHCII^low^ macrophages, an exciting hypothesis that requires further investigation.

Our study has limitations. We included a non-ischemic control group with similar demographic and clinical risk factors as patients with PAD to ensure that we detect PAD-specific differences rather than comorbidity-related effects. Patients with aneurysms have a similar clinical background of hypertension and hyperlipidemia and a similar treatment of statins, antiplatelet therapy and antihypertensive drugs (Table 1). The non-ischemic patients had a normal ABI and did not show claudication symptoms during the walking test, therefore excluding the clinical manifestation of PAD, and did not show occluded vessels as reported by GLASS stage. Additionally, patients with popliteal aneurysm underwent a similar surgical procedure, which allowed us to standardize sample harvesting. We are confident that this strategy dissected differences between both groups as evidenced by the enrichment of gene sets related to abnormal muscle physiology. This said, we cannot exclude that vascular aneurysms affected the composition and genetic fingerprint of the non-ischemic samples. Our patient cohort was relatively small and, therefore, may be subjected to some inadvertent selection bias. Our study included only male participants, so potential sex-based differences could not be assessed.

In conclusion, we used scRNA-seq to characterize the composition and transcriptional changes of the muscle microenvironment in ischemic PAD compared to non-ischemic comorbidity-matched patients. We identified profound transcriptomic alterations in ECs promoting EC dysfunction features (such as EndoMT) while acquiring a more pro-inflammatory phenotype. *LYVE1*^hi^MHCII^low^ macrophages are the main macrophage population in ischemic PAD muscle and get activated during the disease, a phenotype that may be driven by ECs. We propose that unraveling the impaired cellular crosstalk in PAD muscle could potentially offer novel targets for therapeutic intervention.

## Methods

### Patients and study design

Non-ischemic patients and patients with PAD were recruited from the Department of Angiology, Cantonal Hospital Baden in Baden, Switzerland. The general inclusion criteria for both non-ischemic patients and patients with PAD were as follows: scheduled for surgery (bypass surgery in patients with PAD or lower limb aneurysm surgery in non-ischemic samples); age ≥45 years; ability to understand German; and signed informed consent to participate in the study. Patients received no compensation. Inclusion criteria specific for patients with PAD were as follows: diagnosis of PAD ranging from exercised limited claudication (Fontaine classification IIa or IIb) to CLI (Fontaine classification III–IV); ABI ≤ 0.90 at rest unless diagnosed with media sclerosis; and stable medicament regimen (that is, statin, antiplatelet or antihypertensive regimen). Inclusion criteria specific for non-ischemic patients were as follows: diagnosis of lower limb aneurysm and ABI > 1 at rest. The general exclusion criteria for both non-ischemic patients and patients with PAD were as follows: severe peripheral neuropathy (Total Neuropathy Score ≥ 3); unstable angina or severe (>70%) three coronary vessel disease; significant liver dysfunction (Model for End-Stage Liver Disease score) or known liver cirrhosis (Child–Turcotte–Pugh score > 6 points); treated for cancer in the last 2 years; and diagnosis of Parkinson’s disease. Exclusion criterion specific for patients with PAD: above/below-knee amputation. Exclusion criterion specific for non-ischemic patients: peripheral artery disease diagnosed as described before (inclusion criteria specific for patients with PAD). GLASS stage: preoperative angiographic imaging (iodine contrast computed tomography angiography or digital subtraction angiography) was performed. Then, for GLASS stage calculation, the target arterial path was identified, and the femoropopliteal GLASS grade (NA, I, II or III) and the infrapopliteal GLASS grade (NA, I, II or III) were determined. Femoropopliteal and infrapopliteal scores were combined to determine the final GLASS stage. The study was conducted according to the Declaration of Helsinki, the Human Research Act and the Human Research Ordinance, and the protocol was approved by the ethics committee of the Canton of Zurich (KEK number: 2020-01393). Remaining sample material is available upon formal request to the corresponding author (katrien-debock@ethz.ch).

Muscle samples from the peripheral region of the medial head of gastrocnemius muscle (one-third of the distance between the tibial plateau and the malleolus medialis) were collected during lower limb aneurysm surgery (non-ischemic samples) or bypass surgery (PAD samples) and immediately transferred to an ice-cold hypothermic preservation solution (HypoThermosol FRS Preservation Solution; Sigma-Aldrich, H4416) for optimal transport and conservation until further processing. From four PAD and four non-ischemic samples, samples were processed for scRNA-seq analysis, and part of the tissue was also used for histology. A set of samples (*n* = 11 for PAD and *n* = 8 for non-ischemic) was embedded in Optimal Cutting Temperature (OCT) medium, frozen in liquid N_2_-cooled isopentane and stored at −80 °C for histological analysis. Transversal 10-µm cryosections of the samples were prepared at −20 °C, air dried for approximately 30 minutes and stored at −80 °C until further processing. A subset of histology samples (*n* = 3 for PAD and *n* = 3 for non-ischemic) was used for CosMx single-cell spatial transcriptomics (see below). A set of eight independent samples (three non-ischemic and five PAD) was used for isolation and culture of primary muscle ECs (see below).

### Isolation of cells from human skeletal muscle for scRNA-seq

For isolation of cells for scRNA-seq, and in less than 2 hours after collection and preservation in ice-cold hypothermic preservation solution (HypoThermosol FRS Preservation Solution; Sigma-Aldrich, H4416), samples were minced in a Petri dish on ice using scissors and a surgical blade. Minced samples were transferred into a digestion buffer (4 ml of digestion buffer per 100 mg of muscle) containing 2 mg ml^−1^ Dispase II (Sigma-Aldrich, D4693) and 2 mg ml^−1^ Collagenase IV (Thermo Fisher Scientific, 17104019) in HBSS buffer (Thermo Fisher Scientific, 14025100) at 37 °C for 25–30 minutes, with gentle shaking every 3–5 minutes. After digestion, the reaction was stopped by adding an equal volume of 20% heat-inactivated FBS (Thermo Fisher Scientific, 10500064) and 1 mM EDTA (VWR Life Science, E177) in HBSS (Thermo Fisher Scientific, 14025100), and the suspension was passed through a 100-µm cell strainer (Bioswisstec, 800100) and a 40-µm cell strainer (Falcon, 352340). Cell suspension was centrifuged at 500*g* for 5 minutes at 4 °C, and the supernatant was discarded. The pellet was resuspended in 1 ml of ACK Lysing Buffer (Thermo Fisher Scientific, A1049201) for red blood cell lysis for 2 minutes at room temperature. After incubation, the reaction was stopped with 4 ml of 10% heat-inactivated FBS (Thermo Fisher Scientific, 10500064) in HBSS buffer (Thermo Fisher Scientific, 14025100). Cell suspension was centrifuged at 500*g* for 5 minutes at 4 °C, and the pellet was resuspended in 0.5% BSA (PanReac AppliChem, A1391) in DPBS (Thermo Fisher Scientific, 14190250) containing 1 mM EDTA (VWR Life Science, E177) for FACS. Calcein Violet (Thermo Fisher Scientific, 65-0854-39) for metabolically active live cell selection was added directly to the cell suspension at 1:1,000 dilution 10 minutes before sorting.

### Human scRNA-seq analysis

Metabolically active (Calcein^+^) human muscle mononuclear cells were FACS sorted and loaded into a 10x Chromium Next GEM Chip G, aiming for the recovery of approximately 10,000 cells per sample. scRNA-seq libraries were generated according to 10x Chromium Next Gem Single Cell 3′ Reagent Kits version 3.1 (CG000204 Rev D) and sequenced on an Illumina NovaSeq 6000 system. We sequenced a total of 13 samples from eight different patients (Table 1). Sequencing reads were aligned to the human genome using Cell Ranger versions 5.0.0–7.0.0 and the genome assembly GENCODE GRCh38.p13 Annotation Release 37. Intron reads were not included during the alignment. Each individual sample was independently processed for quality control and log normalization using the scran 1.24.1 and scater 1.24.0 packages in R (version 4.2.0). High-quality cells were selected based on library size, number of detected genes and percentage of reads mapped to mitochondrial genes, independently adjusting the thresholds for each sample. Raw unique molecular identifier (UMI) counts were log normalized based on library size factors. Doublets were detected and removed in every individual sample using the default parameters of the function runDoubletFinder from the singleCellTK 2.6.0 package. After individual quality control and log normalization, all samples were batch corrected and merged using the Harmony 0.1.1 package. Dimensionality reduction by t-distributed stochastic neighbor embedding (t-SNE) was performed based on the dimensions generated by Harmony integration. Clustering was performed by building a nearest neighbor graph (*k* = 20) and then applying the Louvain algorithm as implemented in the Seurat 4.1.1 package. For individual analysis and reclustering of the EC, MoMac and SMC/pericyte populations, cells were selected by tracing back the clustering of the complete dataset in every individual sample. Raw counts from selected cells—ECs, MoMacs and SMCs/pericytes—were log normalized in every individual sample and batch corrected and merged as described above for the complete dataset. All dot plots showing gene expression were generated using the plotDots() function as implemented in the scater 1.24.0 package. Cell type proportions in each condition were calculated by dividing the number of cells in each cluster by the total number of cells in that respective condition.

DEGs were detected by performing a Wilcoxon rank-sum test as implemented in the Seurat 4.1.1 package. DEGs were selected based on a minimum log_2_ fold change of 0.25 and an adjusted *P* < 0.05. For ORA, selected DEGs were ordered based on increasing adjusted *P* value and given as an input in the web-based implementation of g:Profiler^26^, selecting the option ‘Ordered query’ before running the analysis. ORA results were represented as the −log_10_ of the *P* values obtained for each process. ORA results were simplified by using the rrvgo 1.10.0 package. Redundant terms were grouped, and only the most significant term of each group was conserved. Schematic visualization of Kyoto Encyclopedia of Genes and Genomes (KEGG) pathways from ORA results was generated using the pathview 1.38.0 package.

For pseudobulk analysis, lowly expressed genes were removed if they were not detected in at least 10 cells. Raw UMI counts were aggregated per patient (total sum of counts for every gene from all cells in every patient). For differential expression analysis in the pseudobulk dataset, we applied the DESeq2 1.38.3 package and performed the comparison between non-ischemic patients and patients with PAD. All significant DEGs (adjusted *P* < 0.05) were reported in a heatmap (Fig. 1j) by using the normalized counts (by size factors) in each patient and scaled and centered based on gene expression per patient. For GSEA in the pseudobulk dataset, we applied the fgsea 1.24.0 package. From the differential expression analysis results performed with DESeq2, we selected the ‘stat’ value (Wald statistic: the log_2_ fold change divided by the standard error of the log fold change (lfcSE)) for each gene and performed GSEA analysis using the default settings of the fgsea() function from the fgsea 1.24.0 package. Significant pathways were selected based on an adjusted *P* < 0.05. Human hallmark pathways from the Molecular Signatures Database (MSigDB)^94^ were used for this analysis.

GSVA was performed using the GSVA 1.46.0 package. Log-normalized counts from the corresponding scRNA-seq dataset (either ECs or MoMacs) were given as an input for the gsva() function as implemented in the GSVA 1.46.0 package. The gene sets to perform GSVA were obtained from Fan et al.^24^ (ATF4-dependent genes as defined by genes with log fold change < −0.1 and adjusted *P* < 0.05 after comparing mouse muscle ECs in wild-type and EC-specific inducible ATF4 knockout mice) or Sanin et al.^48^ (macrophage activation processes). For GSVA of M1–M2 signatures, we obtained the gene sets from Varga et al.^50^, which reported a set of M1–M2 genes derived from in vitro stimulation and generated gene sets for pro-inflammatory (Ly6C^+^) and pro-regenerative (Ly6C^−^) macrophages at different timepoints after cardiotoxin-induced muscle injury.

Bulk RNA-seq data from muscle ECs in wild-type and EC-specific ATF4 knockout mice were obtained from Fan et al.^24^ and processed as described by the authors. Only wild-type and ATF4 knockout red muscle ECs were used in this study.

Transcription factor inference analysis was performed using the Python implementation of the SCENIC package (pySCENIC)^30^ and following the script provided by the authors in their GitHub repository (https://github.com/aertslab/pySCENIC). Cell communication analysis on the complete dataset (all cell types in non-ischemic and PAD samples) was performed using the CellChat 1.6.1 package^53^ following the script provided by the authors in their GitHub repository (‘Full tutorial for comparison analysis of multiple datasets’ section in https://github.com/sqjin/CellChat). Cell communication analysis between *ATF3/ATF4*^+^ ECs and *LYVE1*^hi^MHCII^low^ macrophages was performed using the nichenetr 1.1.0 package^64^ following the script provided by the authors in their GitHub repository (‘Differential NicheNet analysis between conditions of interest’ section in https://github.com/saeyslab/nichenetr).

All raw and processed (10x Cell Ranger output files) sequencing data are available at GSE235143.

### Isolation and culture of ECs from human skeletal muscle

We isolated muscle ECs from eight independent samples (three non-ischemic and five PAD) using an established protocol^95^, yielding high-purity (>95%) EC cultures (Extended Data Fig. 4g). As recommended in the original publication^95^, we performed several rounds of CD31^+^ magnetic beads sorting until we achieved the desired purity. Cells were cultured in EGM-2 MV Microvascular Endothelial Cell Growth Medium-2 BulletKit (Lonza, CC-3202). Subsequently, confluent EC cultures from three patients with PAD were treated for 24 hours with vehicle (DMSO; Carl Roth, A994.1) or celastrol (Sigma-Aldrich, C0869) (125 nM or 250 nM), doses that are consistent with literature and do not affect cell viability of human umbilical vein endothelial cells (HUVECs)^38^. Celastrol powder was dissolved in DMSO at 2 mg ml^−1^ (stock concentration) and later diluted to experimental doses (125 nM or 250 nM) directly into EGM-2 media right before use. Control samples (vehicle) were given the same volume of DMSO as used to dilute celastrol in the 250 nM condition. After 24-hour incubation, RNA was extracted for bulk RNA-seq using an RNeasy Plus Micro Kit (Qiagen, 74034) according to the manufacturer’s protocol. RNA integrity was assessed by using an Agilent High Sensitivity RNA ScreenTape system. Only samples with RNA integrity number ≥ 8.0 were further processed.

For conditioned media experiments, confluent ECs were cultured for 48 hours with fresh EGM-2 medium. Then, media were collected, filtered through a 0.2-µm filter (Sarstedt, 83.1826.001) and cryopreserved at −80 °C until further use. Cells were routinely cultured at 37 °C in 21% O_2_ and 5% CO_2_.

### Macrophage polarization in vitro using conditioned media

In vitro macrophage polarization using conditioned media was performed with an adapted protocol from George et al.^96^. Peripheral blood mononuclear cells were obtained from buffy coats (Swiss Blood Donation Center of Lugano) by density gradient centrifugation using Ficoll-Paque PLUS (Cytiva, 17-1440-02). CD14^+^ cells were isolated using human CD14 MicroBeads (Miltenyi Biotec, 130-050-201) following the manufacturer’s recommendations. The purity of CD14^+^ cells was assessed by flow cytometry (>98%; except from one donor, >90%) (see antibodies below). In total, 5 × 10^5^ CD14^+^ cells per well were seeded in a 48-well plate (TPP, TPP92148) in 250 µl of varying concentrations (0%, 6.25%, 12.5%, 25%, 50%) of conditioned media (three non-ischemic, five PAD). On day 3, the cells were harvested, stained for flow cytometry and acquired on a BD Fortessa. The following antibodies were used: Human TruStain FcX (used for Fc blocking; BioLegend, 422302), Zombie NI Fixable Viability Kit (dilution 1:500, used for live/dead staining; BioLegend, 423106), CD14 (APC-Cy7, dilution 1:200, used for purity assessment; BioLegend, 325620), CD19 (FITC, dilution 1:20, used for purity assessment; BD Biosciences, 555412), CD3 (BV650, dilution 1:200, used for purity assessment; BioLegend, 300468), CD56 (PC5, dilution 1:50, used for purity assessment; Beckman Coulter, A07789), HLA-DR (BV605, dilution 1:100, used for polarization assessment; BD Biosciences, 562845), CD80 (FITC, dilution 1:50, used for polarization assessment; BD Biosciences, 557226) and CD206 (PE, dilution 1:50, used for polarization assessment; BioLegend, 321106). The data were analyzed using FlowJo version 10 software. The experiment was performed twice with CD14^+^ cells from three buffy coats, respectively (total of six different donors).

### Bulk RNA-seq of celastrol-treated ECs

Libraries were generated using a TruSeq Stranded mRNA Library Kit (Illumina) and sequenced on an Illumina NovaSeq X Plus. Sequencing reads were processed using Kallisto and the human genome assembly GENCODE GRCh38.p13 Annotation Release 42 to generate a count file matrix for each individual sample. Samples were pooled together into a single matrix and analyzed following the DESeq2 pipeline. Differential expression analysis was performed after correcting for patient and sequencing plate effect. Gene signatures for GSEA were obtained from Fan et al.^24^ (ATF4; see the ‘Human scRNA-seq analysis’ subsection) and Alhusban et al.^39^ (glucosamine, after analyzing bulk RNA-seq data from the original publication, HUVECs treated with glucosamine (5 mM) versus L-glucose (5 mM) for 6 hours under hypoxia and serum starvation conditions).

Raw and processed (Kallisto aligned) sequencing files are available at GSE287300.

### CosMx single-cell spatial transcriptomics of muscle tissue

Transversal 10-µm cryosections of the samples were prepared at −20 °C, air dried for approximately 30 minutes and shipped to NanoString Technologies under optimal storage conditions using dry ice to ensure sample integrity. Samples were processed by NanoString for CosMx spatial molecular imaging through the Technology Access Program (project ID: SMI0364). For transcript detection, CosMx Human Universal Cell Characterization Panel (RNA, 1000 Plex; CMX-H-USCP-1KP-R) was used. Cell detection (cell segmentation) was performed using a combination of DAPI (nuclei) staining and laminin staining, which marks the basal lamina surrounding individual muscle fibers to provide additional structural context and facilitate accurate segmentation of mononuclear cells located between fibers. Data processing was performed using the Giotto package. Each sample was individually processed for quality control and log normalization and then integrated together using Harmony, following the instructions from Giotto developers (https://github.com/drieslab/Giotto). To integrate spatial samples with scRNA-seq data and transfer cluster labels, spatial samples were treated as scRNA-seq-like datasets (cell-by-gene expression matrices). These were integrated with scRNA-seq samples using the same Harmony pipeline described in the ‘Human scRNA-seq analysis’ subsection. After integration, all cells were projected into a shared dimensionality reduction space. We then used t-SNE coordinates to identify the 10 closest scRNA-seq cells for each spatial cell, using Euclidean distance: √((*x*_2_ − *x*_1_)^2^ + (*y*_2_ − *y*_1_)^2^). The most frequent cluster label among the 10 nearest scRNA-seq cells was assigned to the corresponding spatial cell. For proximity analysis between *LYVE1*^hi^MHCII^low^ macrophages and EC subtypes, a similar approach was followed. In brief, after transferring cluster labels to the spatial dataset, we identified the nearest EC for each *LYVE1*^hi^MHCII^low^ macrophage based on the shortest Euclidean distance in the spatial coordinates.

### Mice

Inducible EC-specific Atf4 knockout (*Pdgfb*-Cre^ERT2^×*Atf4*^ΔEC/ΔEC^) mice were generated as described previously^24^. Recombination was induced in 8–10-week-old mice by daily intraperitoneal administration of 1 mg of tamoxifen (Sigma-Aldrich, T5648) dissolved in 1:10 ethanol:corn oil solution for five consecutive days. A washout period of at least 7 days was allowed before starting the experiments. Tamoxifen-treated Cre-negative littermates were used as control for all experiments. All mice were maintained on a C57BL/6N background. To label proliferating cells, an intraperitoneal injection of 5-ethynyl-2′-deoxyuridine (EdU) (Thermo Fisher Scientific, E10187) solution (5 mg ml^−1^ in saline) was performed 7 hours before euthanizing the mice.

Mice were randomly allocated to different treatment groups, and the investigator was blinded to the group allocation during the experiment as well as during the analysis. Mice were housed at standard housing conditions (22 °C, 12-hour light/dark cycle), with ad libitum access to chow diet (18% protein, 4.5% fiber, 4.5% fat, 6.3% ashes; Provimi Kliba SA) and water. Health status of all mice was regularly monitored according to Federation of European Laboratory Animal Science Associations guidelines. All animal experiments were approved by the local animal ethics committee (Kantonales Veterinärsamt Zürich, license ZH050/2021) and performed according to local guidelines (TschV, Zurich) and the Swiss animal protection law (TschG). All mice in this study were male.

### HLI model

HLI experiments were performed as described previously^42^. Mice were anesthetized with isofluorane; the hindlimbs were shaved; and, after a small incision in the skin, both the proximal end of the femoral artery and the distal portion of the saphenous artery were ligated. The artery and all side-branches were dissected free; after this, the femoral artery and attached side-branches were excised. Immediately after surgery, perfusion was measured by laser Doppler imaging of plantar regions of interest (Moor Instruments, Ltd.) and calculated as ratio of left (ligated) versus right (unligated) values. Perfusion was measured again at different timepoints following the same procedure.

### Tissue immunofluorescence and histology

Human muscles were harvested and embedded in OCT embedding matrix (Carl Roth, 6478.1) and frozen in liquid N_2_-cooled isopentane. Frozen sections (10 µm) were made using a cryostat (Leica, CM1950). The following antibodies were used: anti-LYVE1 (1:200 dilution; Abcam, ab33682), anti-CD68 (1:100 dilution; Abcam, ab955), anti-CD31 (1:200 dilution; Dako, M082329-2), anti-fibronectin (1:1,000 dilution; Abcam, ab23750), goat anti-rabbit IgG cross-adsorbed secondary antibody, Alexa Fluor 568 (1:200 dilution; Thermo Fisher Scientific, A-11001), goat anti-mouse IgG cross-adsorbed secondary antibody, Alexa Fluor 488 (1:200 dilution; Thermo Fisher Scientific, A-1101) and Alexa Fluor 647-conjugated wheat germ agglutinin (WGA, 1:50 dilution; Thermo Fisher Scientific, W32466). Muscle sections were fixed in 4% paraformaldehyde followed by epitope retrieval using sodium citrate (10 mM, pH 6.5) at 92 °C for 20 minutes. Endogenous peroxidase activity was blocked with 3% hydrogen peroxide in PBS followed by 10 minutes of permeabilization (PBS + 0.5% Triton X-100) and 60 minutes of blocking at room temperature in blocking buffer (PBS + 1% BSA). After blocking, sections were stained overnight at 4 °C with primary antibodies, followed by 1-hour incubation of secondary antibodies at room temperature. ECM was stained using WGA. Nuclei were detected using Hoechst diluted 1:5,000 in PBS and for 5 minutes at room temperature. Slides were then washed with PBS and mounted using Immu-Mount (Thermo Fisher Scientific, 9990412). Slides were imaged on an AxioObserver.Z1 fluorescence microscope using ×20 and ×40 objectives (Zeiss). For the LYVE1 and CD68 staining, 315 ± 106 (mean ± s.d.) fibers were counted per patient, and, for the CD31 staining, 409 ± 80 (mean ± s.d.) fibers were counted. Different regions over the whole tilescan muscle section were analyzed with ZEN software (ZEN 2011 imaging software; Zeiss) and Fiji. General tissue morphology was evaluated using hematoxylin and eosin (H&E) staining. H&E images were acquired with an Eclipse Ti2 inverted microscope (Nikon) using a ×20 objective.

Mouse muscles were harvested and embedded in OCT embedding matrix (Carl Roth, 6478.1) and frozen in liquid N_2_-cooled isopentane. Frozen sections (10 µm) were made using a cryostat (Leica, CM1950). For EdU detection combined with CD31, EdU was first visualized using the EdU Click-iT Cell Reaction Buffer Kit (Thermo Fisher Scientific, C10269) according to the manufacturer’s instructions and subsequently incubated for 1 hour in blocking buffer (PBS with 1% BSA) at room temperature. Thereafter, sections were incubated overnight at 4 °C with goat anti-mouse/rat CD31/PECAM-1 antibody (1:250; R&D Systems, 3628) diluted in blocking buffer with 0.1% Triton X-100.

### Statistics

The histology images presented in this paper are representative of the data (quantification of images is approximately the group average) and the staining quality. All human imaging data are represented as violin plots, where the three horizontal lines represent the median and quartiles, and the width of the plot indicates the distribution of the data. GraphPad Prism software (version 9.2.0) was used for statistical analyses. The Shapiro–Wilk test was performed to analyze data distribution. Non-normally distributed data were analyzed by non-parametric Mann–Whitney *U*-test to compare two groups. Normally distributed data were analyzed using Student’s *t*-test in an unpaired two-tailed fashion to compare two groups. For experiments evaluating more than one variable, a two-way ANOVA with Tukey’s multiple comparison was used. For experiments evaluating more than one variable with a repeated-measures design, a two-way ANOVA with Sidakʼs multiple comparison test was used. Each figure legend indicates the statistical approach for each experiment displayed in the figure. *P* > 0.05 was considered non-significant. Asterisks in figures denote statistical significance. R and related packages were used for RNA-seq statistical analysis. Unless otherwise indicated, the default statistical test (for example, Wilcoxon rank-sum test for differential expression) from each package function was used. No animals or data points were excluded from the analysis.

### Reporting summary

Further information on research design is available in the Nature Portfolio Reporting Summary linked to this article.

## Supplementary information

Reporting Summary

Supplementary Data 1 File containing additional data on corresponding figures.

## Source data

Source Data Fig. 1 Statistical source data.

Source Data Fig. 2 Statistical source data.

Source Data Fig. 4 Statistical source data.

Source Data Fig. 7 Statistical source data.

Source Data Extended Data Fig./Table 1 Statistical source data.

Source Data Extended Data Fig./Table 2 Statistical source data.

Source Data Extended Data Fig./Table 3 Statistical source data.

Source Data Extended Data Fig./Table 4 Statistical source data.

Source Data Extended Data Fig./Table 5 Statistical source data.

## Data Availability

The RNA-seq data underlying this article can be fully explored at https://shiny.debocklab.hest.ethz.ch/Turiel-et-al/. Additionally, raw data are available in the Gene Expression Omnibus at https://www.ncbi.nlm.nih.gov/geo/ and can be accessed with accession numbers GSE235143 (scRNA-seq) and GSE287300 (celastrol bulk RNA-seq). Processed data (scRNA-seq datasets after quality control, normalization, integration and clustering) can be accessed at figshare (10.6084/m9.figshare.29493215.v1)^97^.

## Code availability

The following codes were used: SCENIC (https://github.com/aertslab/pySCENIC), CellChat (https://github.com/sqjin/CellChat), NicheNet (https://github.com/saeyslab/nichenetr) and Giotto (https://github.com/drieslab/Giotto). Any additional code required to reproduce the results from this paper is available from the corresponding author upon reasonable request (katrien-debock@ethz.ch).
