# Supplementary material for: Single-cell compendium of muscle microenvironment in peripheral artery disease reveals altered endothelial diversity and LYVE1+ macrophage activation
Source: Nat Cardiovasc Res. 2025 Sep 15;4(10):1221–40. doi: 10.1038/s44161-025-00709-y (PMC12520974; doi:10.1038/s44161-025-00709-y)
Supplement: Supplementary file 1 — Reporting Summary [file 44161_2025_709_MOESM1_ESM.pdf]

nature portfolio

Corresponding author(s): Katrien De Bock

Last updated by author(s): Aug 9, 2025

Reporting Summary

Nature Portfolio wishes to improve the reproducibility of the work that we publish. This form provides structure for consistency and transparency in reporting. For further information on Nature Portfolio policies, see our Editorial Policies and the Editorial Policy Checklist.

Statistics

For all statistical analyses, confirm that the following items are present in the figure legend, table legend, main text, or Methods section.

n/a	Confirmed	
☐	☒	The exact sample size (*n*) for each experimental group/condition, given as a discrete number and unit of measurement
☐	☒	A statement on whether measurements were taken from distinct samples or whether the same sample was measured repeatedly
☐	☒	The statistical test(s) used AND whether they are one- or two-sided<br>*Only common tests should be described solely by name; describe more complex techniques in the Methods section.*
☒	☐	A description of all covariates tested
☐	☒	A description of any assumptions or corrections, such as tests of normality and adjustment for multiple comparisons
☐	☒	A full description of the statistical parameters including central tendency (e.g. means) or other basic estimates (e.g. regression coefficient) AND variation (e.g. standard deviation) or associated estimates of uncertainty (e.g. confidence intervals)
☐	☒	For null hypothesis testing, the test statistic (e.g. *F*, *t*, *r*) with confidence intervals, effect sizes, degrees of freedom and *P* value noted<br>*Give P values as exact values whenever suitable.*
☒	☐	For Bayesian analysis, information on the choice of priors and Markov chain Monte Carlo settings
☒	☐	For hierarchical and complex designs, identification of the appropriate level for tests and full reporting of outcomes
☒	☐	Estimates of effect sizes (e.g. Cohen's *d*, Pearson's *r*), indicating how they were calculated

Our web collection on statistics for biologists contains articles on many of the points above.

Software and code

Policy information about availability of computer code

Data collection	Fluorescence imaging: ZEN software (Zeiss).
Data analysis	Bioinformatic analysis: CellRanger v5.0.0-7.0.0, Kallisto, R 4.2.0, R packages: scater 1.24.0, scran 1.24.1, singleCellTK 2.6.0 , harmony 0.1.1, Seurat 4.1.1, rrvgo 1.10.0, pathview 1.38.0, SingleR 1.8.0, DESeq2 1.38.3, fgsea 1.24.0, GSVA 1.46.0, pySCENIC, CellChat 1.6.1, nichenetr 1.1.0, Giotto, clusterProfiler 4.2.0. Image analysis: ImageJ 1.53. FACS: FlowJo v10. Statistical analysis: GraphPad Prism 9.

For manuscripts utilizing custom algorithms or software that are central to the research but not yet described in published literature, software must be made available to editors and reviewers. We strongly encourage code deposition in a community repository (e.g. GitHub). See the Nature Portfolio guidelines for submitting code & software for further information.

Data

Policy information about availability of data

All manuscripts must include a data availability statement. This statement should provide the following information, where applicable:

- Accession codes, unique identifiers, or web links for publicly available datasets
- A description of any restrictions on data availability
- For clinical datasets or third party data, please ensure that the statement adheres to our policy

The RNA sequencing data underlying this article can be fully explored at https://shiny.debocklab.hest.ethz.ch/Turiel-et-al/. Additionally, raw data are available in Gene Expression Omnibus (GEO) at https://www.ncbi.nlm.nih.gov/geo/ and can be accessed with accession number GSE235143 (scRNAseq) and GSE287300

(celastrol bulk RNAseq). Processed data (scRNAseq datasets after QC, normalization, integration and clustering) can be accessed at Figshare (https://doi.org/10.6084/m9.figshare.29493215).

Research involving human participants, their data, or biological material

Policy information about studies with human participants or human data. See also policy information about sex, gender (identity/presentation), and sexual orientation and race, ethnicity and racism.

Reporting on sex and gender	Patients from both sexes were allowed to participate in the study but eventually we were only able to recruit males. Potential disaggregations for sex and gender were not collected. Sex and gender of participants was based on self-report.
Reporting on race, ethnicity, or other socially relevant groupings	N/A. racial/ethnic identity was not collected.
Population characteristics	Patient characteristics are provided in Table 1
Recruitment	Non-ischemic and PAD patients were recruited from the Department of Angiology, Cantonal Hospital Baden, Baden, Switzerland (Stephan Engelberger, MD) based on the recruitment criteria (outlined in methods section). All patients signed an informed consent before participating in the study. Patients received no compensation.
Ethics oversight	The study was conducted according to the Declaration of Helsinki, the Human Research Act (HRA) and the Human Research Ordinance (HRO) and the protocol was approved by the Ethics Committee of the Canton of Zurich (KEK number: 2020-01393).

Note that full information on the approval of the study protocol must also be provided in the manuscript.

Field-specific reporting

Please select the one below that is the best fit for your research. If you are not sure, read the appropriate sections before making your selection.

☒ Life sciences ☐ Behavioural & social sciences ☐ Ecological, evolutionary & environmental sciences

For a reference copy of the document with all sections, see nature.com/documents/nr-reporting-summary-flat.pdf

Life sciences study design

All studies must disclose on these points even when the disclosure is negative.

Sample size	No sample-size calculations were performed. Sample size was determined to be adequate based on the magnitude and consistency of measurable differences between groups.
Data exclusions	Grubbs test was used to determine significant outliers (Alpha = 0.05).
Replication	Number of replicates are explained in the figure legends.
Randomization	Human samples were allocated to experimental groups (non-ischemic or PAD) based on previous clinical diagnosis. Both groups were co-morbidity matched. Mice were randomly allocated to different treatment groups, and the investigator was blinded to the group allocation during the experiment as well as during the analysis. Tamoxifen-treated Cre-negative littermates were used as control for all experiments.
Blinding	Bioinformatic analysis: investigators were not blinded during bioinformatic analyses as human samples were previously diagnosed to organize sample collection and processing. Histological analysis: investigators were blinded during the experiments and analysis. In vivo and in vitro experiments: investigators were blinded during the experiments and analysis.

Reporting for specific materials, systems and methods

We require information from authors about some types of materials, experimental systems and methods used in many studies. Here, indicate whether each material, system or method listed is relevant to your study. If you are not sure if a list item applies to your research, read the appropriate section before selecting a response.

Materials & experimental systems

n/a	Involved in the study	
☐	☒	Antibodies
☒	☐	Eukaryotic cell lines
☒	☐	Palaeontology and archaeology
☐	☒	Animals and other organisms
☒	☐	Clinical data
☒	☐	Dual use research of concern
☒	☐	Plants

Methods

n/a	Involved in the study	
☒	☐	ChIP-seq
☒	☐	Flow cytometry
☒	☐	MRI-based neuroimaging

Antibodies

Antibodies used

Histology: anti-LYVE1 (1:200 dilution, ab33682, Abcam), anti-CD68 (1:100 dilution, ab955, Abcam), anti-CD31 (1:200 dilution, M082329-2, Dako), anti-Fibronectin (1:1000 dilution, ab23750, Abcam), Goat anti-Rabbit IgG Cross-Adsorbed Secondary Antibody, Alexa Fluor 568 (1:200 dilution, A-11001, Thermofisher), Goat anti-Mouse IgG Cross-Adsorbed Secondary Antibody, Alexa Fluor 488 (1:200 dilution, A-1101, Thermofisher), Alexa Fluor 647 conjugated wheat germ agglutinin (WGA, 1:50 dilution, W32466, ThermoFisher).
Macrophage polarization: Human TruStain FcX (used for Fc blocking; 422302, BioLegend), Zombie NIR™ Fixable Viability Kit (dilution 1:500, used for Live/Dead staining; 423106, BioLegend), CD14 (APC-Cy7, dilution 1:200, used for purity assessment; 325620, BioLegend), CD19 (FITC, dilution 1:20, used for purity assessment; 555412, BD Biosciences), CD3 (BV650, dilution 1:200, used for purity assessment; 300468, BioLegend), CD56 (PC5, dilution 1:50, used for purity assessment; A07789, Beckman Coulter), HLA-DR (BV605, dilution 1:100, used for polarization assessment; 562845, BD Biosciences), CD80 (FITC, dilution 1:50, used for polarization assessment; 557226, BD Biosciences), and CD206 (PE, dilution 1:50, used for polarization assessment; 321106, BioLegend).

Validation

Single color samples and negative controls (no secondary antibody or fluorescence minus one controls) were used for all antibodies.

Animals and other research organisms

Policy information about studies involving animals; ARRIVE guidelines recommended for reporting animal research, and Sex and Gender in Research

Laboratory animals

Mus musculus, Inducible EC-specific Atf4 knockout (Pdgfb-CreERT2 x Atf4ΔEC/ΔEC) on a C57BL/6N background, 8-10 weeks old mice.

Wild animals

The study did not involve wild animals.

Reporting on sex

All mice in this study were male.

Field-collected samples

The study did not involve samples collected from the field.

Ethics oversight

All animal experiments were approved by the local animal ethics committee (Kantonales Veterinärsamt Zürich, license ZH050/2021), and performed according to local guidelines (TschV, Zurich) and the Swiss animal protection law (TschG).

Note that full information on the approval of the study protocol must also be provided in the manuscript.
